# Human Peripheral Blood Gamma Delta T Cells: Report on a Series of Healthy Caucasian Portuguese Adults and Comprehensive Review of the Literature

**DOI:** 10.3390/cells9030729

**Published:** 2020-03-16

**Authors:** Sónia Fonseca, Vanessa Pereira, Catarina Lau, Maria dos Anjos Teixeira, Marika Bini-Antunes, Margarida Lima

**Affiliations:** 1 Laboratory of Cytometry, Unit for Hematology Diagnosis, Department of Hematology, Hospital de Santo António (HSA), Centro Hospitalar Universitário do Porto (CHUP), Unidade Multidisciplinar de Investigação Biomédica, Instituto de Ciências Biomédicas Abel Salazar, Universidade do Porto (UMIB/ICBAS/UP); 4099-001 Porto Porto, Portugal; soniafonseca.hematologiaclinica@chporto.min-saude.pt (S.F.); catarinalau.hematologiaclinica@chporto.min-saude.pt (C.L.); mariateixeira.hematologiaclinica@chporto.min-saude.pt (M.d.A.T.); 2 Department of Clinical Pathology, Centro Hospitalar de Vila Nova de Gaia/Espinho (CHVNG/E); 4434-502 Vila Nova de Gaia, Portugal; v_lpereira@hotmail.com; 3 Laboratory of Immunohematology and Blood Donors Unit, Department of Hematology, Hospital de Santo António (HSA), Centro Hospitalar Universitário do Porto (CHUP), Unidade Multidisciplinar de Investigação Biomédica, Instituto de Ciências Biomédicas Abel Salazar, Universidade do Porto (UMIB/ICBAS/UP); 4099-001Porto, Portugal; u08095@chporto.min-saude.pt

**Keywords:** gamma delta T cells, gamma delta T cell repertoire, Vdelta1, Vdelta2, Vgamma9, normal reference values, human herpes viruses, human cytomegalovirus, immune response

## Abstract

Gamma delta T cells (Tc) are divided according to the type of Vδ and Vγ chains they express, with two major γδ Tc subsets being recognized in humans: Vδ2Vγ9 and Vδ1. Despite many studies in pathological conditions, only a few have quantified the γδ Tc subsets in healthy adults, and a comprehensive review of the factors influencing its representation in the blood is missing. Here we quantified the total γδ Tc and the Vδ2/Vγ9 and Vδ1 Tc subsets in the blood from 30 healthy, Caucasian, Portuguese adults, we characterized their immunophenotype by 8-color flow cytometry, focusing in a few relevant Tc markers (CD3/TCR-γδ, CD5, CD8), and costimulatory (CD28), cytotoxic (CD16) and adhesion (CD56) molecules, and we examined the impacts of age and gender. Additionally, we reviewed the literature on the influences of race/ethnicity, age, gender, special periods of life, past infections, diet, medications and concomitant diseases on γδ Tc and their subsets. Given the multitude of factors influencing the γδ Tc repertoire and immunophenotype and the high variation observed, caution should be taken in interpreting “abnormal” γδ Tc values and repertoire deviations, and the clinical significance of small populations of “phenotypically abnormal” γδ Tc in the blood.

## 1. Introduction

Gamma delta T cells (γδ Tc) are a minor Tc population in the peripheral blood (PB) from human adults [1,2,3]. They are usually divided according the type of Vδ chain they express at the T cell receptor (TCR), with two major γδ Tc subsets being relatively well characterized in humans in terms of tissue distribution, antigen (Ag) recognition patterns and functional properties: Vδ2 and Vδ1 Tc. Vδ2 chain associates with Vγ9 in most cases, defining a Vγ9Vδ2 Tc population that is unique to humans and other primates, whereas Vδ1 Tc use diverse Vγ regions [4,5,6].

Gamma delta Tc is involved in the immune response to viruses, intracellular bacteria, and parasitic protozoa [7,8,9,10,11,12,13,14]; is involved in immune surveillance against hematological neoplasms and solid tumors [15,16,17] and the pathogenesis of autoimmune diseases [18,19]; and abnormal percentages and/or absolute numbers of γδ Tc involving different γδ Tc subsets have been reported in the PB and in different organs from patients with various pathological conditions. Vδ2 Tc respond mainly to intracellular bacteria and solid tumors, while Vδ1 Tc, resident mainly in tissues, is used mostly in the defense against viruses and malignancies, although this dichotomy is not absolute. In addition, γδ T-cells are being explored in cell-based immunotherapy [20,21,22].

In parallel to the low frequency of γδ Tc, lymphoproliferative disorders (LPD) of γδ Tc are rare disease conditions. These comprise γδ Tc large granular lymphocyte (LGL) proliferations, including γδ Tc LGL leukemia, defined as clonal expansions of cytotoxic γδ Tc, which have an indolent clinical course and often coexist with cytopenias and other pathologies [23,24], and hepatosplenic and other γδ Tc lymphomas, which usually have an aggressive clinical course, poor response to chemotherapy and short survival [25,26,27,28].

Gamma delta Tc expressing Vγ9Vδ2 are known to identify microbe-derived (e.g., (E)-4-hydroxy-3-methyl-but-2-enyl pyrophosphate, HMB-PP) and host-derived (e.g., isopentenyl pyrophosphate, IPP) phosphorylated metabolites (“phosphoantigens”) originating from the isoprenoid metabolic mevalonate and non-mevalonate pathways, through association with butyrophilin 3A1 (B3A1) [29,30]. Vδ1 Tc are mainly located in the epithelia, where they interact with cells expressing stress-induced ligands through interaction with natural killer group 2 member D (NKG2D) activating receptors [31,32]. The former includes major histocompatibility complex (MHC) class I polypeptide-related chains (MIC) A and B, and UL16 binding proteins (ULBP), also termed retinoic acid early transcripts (RAET1) [31,32]. Although being first described for Vδ1 Tc, interactions of the ULBP and MIC-A/B molecules with NKG2D are now recognized to also stimulate Vδ2 Tc [31,32]. In addition, Vδ1 Tc recognize lipids and glycolipids presented by CD1d molecules [33,34,35]. Finally, both γδ Tc subsets, Vδ1 and Vδ2, are activated by heat shock proteins (HSP) [36,37], which are upregulated in stressed, infected and malignant cells [4,5,6].

In humans, the size of the circulating pool of γδ Tc is small at birth, and it expands during the first decade of life, from less than 2% of cord blood (CB) Tc to around 5% of PB Tc in adults [38]. Vδ1 Tc, which is the dominant γδ Tc subset at birth, becomes progressively less represented, and the initially small Vγ9Vδ2 subset already predominates by 10 years of age [38]. In the PB from human adults, most γδ Tc expresses Vδ2, usually paired with Vγ9, the remainder being mostly Vδ1, and some Vδ3 or Vδ5, all paired with different Vγ chains [38].

Analysis of PB lymphocyte subsets by flow cytometry (FCM) has become an essential tool in the investigation of different pathological conditions. As such, evaluation of the TCR Vβ, Vγ and Vδ repertoires, using monoclonal antibodies (mAbs) specific for distinct families of the TCR V regions, has been used to study the αβ and γδ Tc compartments in inflammatory, infectious, autoimmune and neoplastic diseases [23,39,40,41,42,43,44,45,46,47]. Additionally, different types of γδ Tc neoplasms have been demonstrated to originate preferentially from distinct Vγ/Vδ Tc subsets [23,40,48]. In accordance, for appropriate diagnosis and follow-up of patients, correct interpretation of the laboratory results from normal reference intervals is required, and more information is needed about the correspondent V usage in healthy individuals. For TCR-Vβ, there have already been a large number of studies performed in normal PB samples [41,49,50]. For TCR-Vγ and Vδ, however, fewer studies have been published so far, and several focused on only one γδ Tc subset [51,52,53,54,55,56].

We report on the percentages and absolute numbers of γδ Tc and Vδ1 and Vδ2/Vγ9 Tc subsets in the PB from a series of 30 Caucasian, Portuguese healthy adults, and the main phenotypic characteristics of these cells, concerning the expression of CD3, TCR, CD5, CD8, CD16, CD28 and CD56 molecules. Additionally, we review the literature on the subject, focusing on the influences of race/ethnicity, age, gender, special periods of life, past immunological experiences, diet, medications and past and concomitant diseases on γδ Tc and their subsets. We emphasize the role of γδ Tc in mediating the immune response in viral infections, and the abilities of different types of viruses, mainly herpesviruses, to activate γδ Tc and modulate γδ Tc repertoires. Finally, using “take-home-messages,” we highlight some key points about γδ Tc we want the reader to remember.

## 2. Materials and Methods

### 2.1. Study Population

A cross-sectional study was conducted with 30 Caucasian Portuguese healthy adults (blood donors, excluding those who were giving blood for the first time), 18 males and 12 females, with a median age of 47 years, ranging from 26 to 66 years (mean ± standard deviation of 48 ± 14 years). All gave informed consent to participate, and the procedures followed the Helsinki Declaration.

#### 2.1.1. Clinical Criteria for Blood Donation

Before blood donation, blood donors were required to fill out an informed consent form and a self-assessment/self-exclusion standardized questionnaire, to ensure that they were in good health, and free of infections and other diseases potentially transmissible by blood; they were also interviewed by a clinician, being submitted to a brief clinical assessment and physical evaluation which included height, blood pressure and capillary hemoglobin levels. Criteria for blood donation followed the Guidelines on Assessing Donor Suitability for Blood Donation recommended by the World Health Organization (WHO), and the Portuguese legislation.

#### 2.1.2. Screening of Blood Transmitted Diseases and Other Blood Tests

As part of routine blood donation procedures, blood donors were tested for human immunodeficiency viruses (HIV) type 1 and 2 (HIV-1 and 2), human T cell lymphoma/leukemia viruses type I and II (HTLV-I and II) and hepatitis B (HBV) and C (HCV) viruses; and for *Treponema Pallidum*, using serological studies. In addition, nucleic acid tests were performed for HIV, HCV and HIV. Alanine aminotransferase serum levels were also determined.

### 2.2. Blood Samples

Peripheral blood samples obtained by venipuncture were collected in BD Vacutainer tubes (Becton Dickinson—BD, San Jose, CA, USA) containing tri-potassium ethylenediaminetetraacetic acid (K3-EDTA) and processed within 6 h of collection.

### 2.3. Blood Cell Counts

Blood cell counts were obtained using a Coulter LH750 automated hematology analyzer (Beckman Coulter—BC, Miami, FL, USA).

### 2.4. Flow Cytometry

#### 2.4.1. Monoclonal Antibodies and Cell Staining

Whole blood was analyzed by ten-parameter eight-color FCM using the following mouse anti-human mAbs from BC/Immunotech (IOT), BD or Endogen: anti-Vδ1 (clone TS8.2; Endogen), anti-Vδ2 (clone Immu389; BC/IOT) or anti-Vγ9 (clone Immu360; BC/IOT), conjugated with fluorescein isothiocyanate (FITC); anti-CD56, conjugated with phycoerythrin (PE) (clone NCAM16.2; BD); anti-TCR-γδ conjugated with PE-Cyanine 5.5 (PC5.5) (clone Immu510; BC/IOT); anti-CD28, conjugated with PE-Cyanine 7 (PC7) (clone CD28.2; BC/IOT); anti-CD5, conjugated with allophycocyanin (APC) (clone L17F12; BD); anti-CD3, conjugated with allophycocyanin cyanine 7 (APC-H7) (clone SK7; BD); anti-CD16, conjugated with violet 450 (V450) (clone 3G8; BD); and anti-CD8, conjugated with krome orange (KO) (clone B9.11; BC/IOT). These mAbs were combined in three distinct tubes, in order to quantify and characterize γδ Tc and their subsets: Vδ1 or Vδ2 or Vγ1/CD56/TCR-γδ/CD28/CD5/CD3/CD16/CD8. According to the manufacturer, the “pan” anti-TCR-γδ mAb used (clone IMMU510) recognizes all γδ Tc, regardless of the variable genes or junction regions they express.

Briefly, 100 μL of PB was incubated for 15 min at room temperature with the appropriated mAbs, subjected to red blood cell lysis and leukocyte fixation using the BD FACS^TM^ lysing solution, washed once, suspended in phosphate buffered saline and then acquired in the flow cytometer.

#### 2.4.2. Flow Cytometer and Sample Acquisition

Sample acquisition was performed in a BD FACSCanto II^TM^ flow cytometer calibrated according to the EuroFlow Standard Operating Procedures [57]. External quality control assessment was achieved by participating in the EuroFlow Quality Assurance program [58]. A median number of 443,388 events was recorded per tube and stored as FCM standard (.fcs) 3.0 files, for subsequent analysis.

#### 2.4.3. Data Analysis

Flow cytometry data files were analyzed using the Infinicyt^TM^ software (Cytognos, Salamanca, Spain). The following percentages were calculated: lymphocytes (among white blood cells, WBC); Tc (among lymphocytes and among WBC); γδ Tc (among total Tc and among WBC); Vδ1, Vδ2 and Vγ9 Tc (among γδ Tc and among WBC); CD5+, CD8+, CD16+, CD28+ and CD56+ Tc (among γδ Tc, and among Vδ1, Vδ2 and Vγ9 Tc). In addition, the median fluorescence intensities (MedFI) of CD3, TCR-γδ, CD5, CD8, CD16, CD28 and CD56 expression were evaluated in each cell population: γδ Tc and αβ Tc (i.e., TCR-γδ negative Tc), and Vδ1, Vδ2 and Vγ9 Tc.

**Figure 1:**
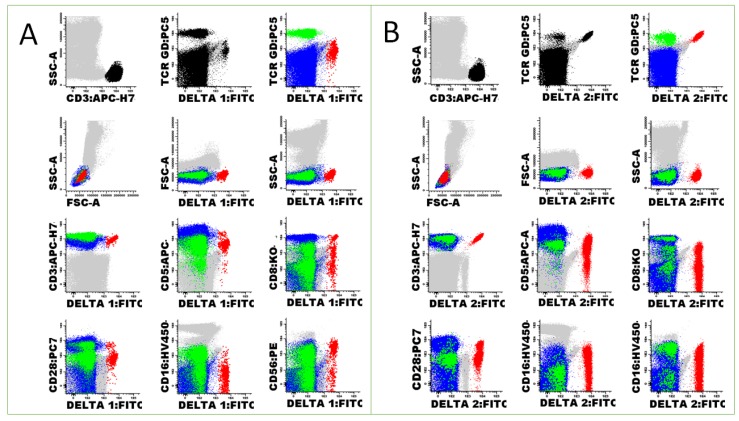
Dot plots obtained from the peripheral blood of a healthy adult stained with an eight-color panel of mAbs, which includes a pan anti-TCR-γδ mAb, anti-Vδ1 (**A**) or an anti-Vδ2 (**B**) mAbs, and mAbs against CD3, CD5, CD8, CD28, CD16 and CD56. Vδ1 Tc (**A**, red dots) represented 5.6% of γδ Tc, 0.9% of Tc and 0.2% of WBC, whereas Vδ2 Tc (**B**, red dots) were 94.4% of γδ Tc, 14.5% of Tc and 2.7% of WBC. Strategy used for γδ Tc gating and analysis: After removing debris and cell aggregates using the SSC-A/FSC-A and the FSC-H/FSC-A dot plots, Tc were identified based on the expression of CD3 (first dot plot in the first row of each panel, black dots), and observed for the staining with anti-TCR γδ and the mAb specific for each of the Vγ or Vδ regions (second dot plot in the first row of each panel, black dots). Afterwards, γδ Tc expressing the putative V chain were painted in red; other γδ Tc were painted in green; and TCR-γδ negative Tc (i.e., αβ Tc) were painted in blue (third dot plot in the first row of each panel). Finally, γδ Tc were analyzed for their FSC and SSC (second row of each panel) and for the expression of cell surface molecules (third and fourth rows of each panel).

The strategy used for cell gating and analysis is depicted in Figure 1.

Absolute counts of total Tc, γδ Tc and Vδ1; Vδ2; and Vγ9 Tc, expressed as numbers of cells/μL, were calculated using a dual platform method, in which total WBC counts were obtained from the hematology analyzer.

### 2.5. Statistics

#### 2.5.1. Statistic Software

Data were entered first into an Excel table and then exported to MedCalc Statistical Software version 16.4.3 (MedCalc Software, Ostend, Belgium) for analysis.

#### 2.5.2. Descriptive Statistics

Descriptive statistics included the absolute and relative frequencies for the qualitative variables, and medians, minimum and maximum values for the quantitative continuous variables. Although several parameters did not have normal distributions (please see below), the mean and standard deviation (SD) values were also calculated in order to facilitate comparisons with other studies.

#### 2.5.3. Statistic Tests

The normality of distributions was assessed using the D’Agostino-Pearson (D’A-PT) and the Kolmogorov-Smirnov (KST) tests. Of the variables tested, the hypothesis of normal distribution was accepted for all the hematological parameters, including the WBC and platelet counts; the hemoglobin levels; and the percentages and absolute numbers of neutrophils and monocytes (Table S1 in Supplementary Materials). Concerning the immunological parameters, a normal distribution was accepted for the percentages of Tc (among lymphocytes) and Tc counts, and for the percentages of Vδ1, Vδ2 and Vγ9 Tc (among γδ Tc). It was rejected for the absolute numbers and percentages of γδ Tc (among Tc); the ratios between Vδ2 and Vδ1 Tc; and the absolute numbers of γδ Tc, including the Vδ1, Vδ2 and Vγ9 Tc subsets (Table S1). As several of the studied variables did not present a normal distribution and given the small size of the sample, non-parametric tests were used for statistics. The coefficients of skewness and kurtosis were used as measures for the degree of symmetry and for the degree of tailedness in the variable distribution.

The Mann-–Whitney U test (MWUT) and the Wilcoxon signed rank test (WSRT) were used to compare continuous variables of two independent and two related samples, respectively. The F-test (FT) was applied to compare the variances. The Spearman rank correlation test (SRCT) was used to evaluate the association between two variables. In addition, linear regression analysis was performed. The Tukey test (TT) was used to check for outliers, which were subsequently excluded when indicated.

*p* values less than 0.05 were considered statistically significant.

#### 2.5.4. Normal Reference Intervals

The National Committee for Clinical Laboratory Standards (NCCLS), and the Clinical and Laboratory Standards Institute (CLSI) guidelines C28-A2 [59] and EP28-A3C [60], were followed for estimating percentiles and their 90% confidence intervals; percentiles were calculated as the observations corresponding to rank r = p*(*n* + 1) and confidence intervals were calculated using integer ranks. The 95th reference interval (reference range) was calculated using three methods [60]: (a) as 97.5th percentile of the ordering data, assuming a normal distribution; (b) using a non-parametrical percentile method, recommended by CLSI for large samples (*n* ≥ 120); and (c) using a “robust method” in which the confidence intervals for the two limits are calculated using the percentile bootstrap method (percentile interval method) recommended by the CLSI for small samples (*n* < 120).

#### 2.5.5. Graphics

Graphics are displayed as box-and-whisker plots in which the central box represents the values from the lower to upper quartile (25th–75th percentile), the middle line represents the median and the horizontal line extends from the minimum to the maximum value, excluding “outliers” which are displayed as separate squares. The outliers were defined as values that were smaller than the lower quartile minus 1.5 times the interquartile range, or larger than the upper quartile plus 1.5 times the interquartile range.

### 2.6. Literature Review

A literature review of the studies published was conducted in the PubMed, to locate potentially relevant, peer-reviewed original and review manuscripts indexed in the MEDLINE database.

## 3. Results

### 3.1. Hematological Counts, Lymphocyte Populations and Gamma Delta T Cells

The median, minimum and maximum; the mean ± SD of the hematological counts and percentages; and the absolute numbers of the PB cell populations analyzed are shown in Table 1. The median percentage of γδ Tc among Tc was of 4.3%, ranging from 1.2% to 15.4%, and the median absolute number of γδ Tc was 63/μL, ranging from 9 to 253 cells/μL (Table 1).

### 3.2. Gamma Delta T cell Immunophenotype

**Figure 2:**
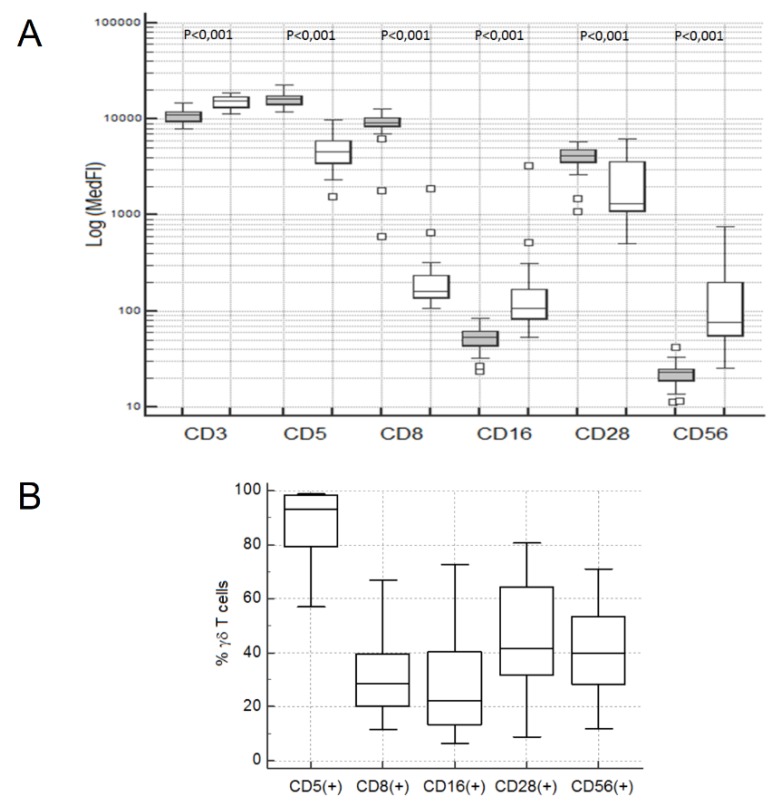
Median fluorescence intensities observed for CD3, CD5, CD8, CD16, CD28 and CD56 molecules on blood αβ (gray boxes) and γδ Tc (white boxes) (**A**), and percentages of CD5+, CD8+, CD16+, CD28+ and CD56+ cells among peripheral blood γδ Tc (**B**) in the study population of healthy adults. *p* values (Mann–Whitney U test) are indicated inside the graphic.

Overall, γδ Tc exhibited higher CD3, CD16 and CD56 levels, and they had lower levels of CD5 and CD28 expression than αβ Tc, as evaluated by the MedFI of each marker in the correspondent Tc population; additionally, the levels of CD8 expression were significantly lower than those observed on CD8 + high αβ Tc (MWUT and WSRT: *p* < 0.001 in all cases) (Figure 2A).

An appreciable fraction of γδ Tc were terminal effector cytotoxic Tc (i.e., LGL), as defined by absence of CD28 expression (58.4%; 19.4–91.0%), concomitantly to the expression of CD16 and/or CD56 in a variable fraction of cells (45.3%; 12.3–77.3%) (Figure 2B and Table S2); in some cases, γδ Tc had high levels of CD16 and/or CD56 expression, suggesting a fully differentiated cytotoxic phenotype, whereas in other cases, low levels of these molecules were observed. CD5 and CD8 were also expressed in a variable fraction of γδ Tc, with median and range values of 93.2% (57.1–98.9%) and 28.6% (11.5–66.9%), respectively (Figure 2B and Table S2).

**Figure 3:**
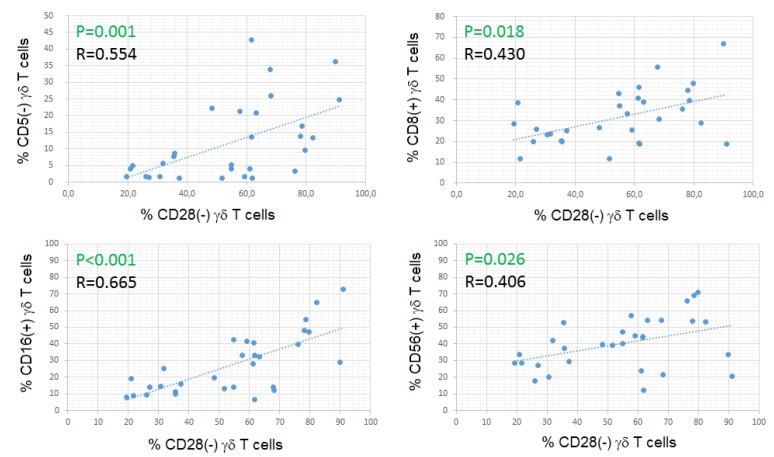
Correlations between the percentages of CD5-, CD8+, CD16+ and CD56+ cells, and the percentages of CD28- γδ Tc in in the study population of healthy adults. The Spearman’s rank correlation coefficients and *p* values are indicated inside the graphics.

The percentage of CD28- cells among γδ Tc (median: 58.4%; range: 19.4–91.1%) was significantly higher than that observed in the αβ Tc compartment (median: 14.0%; range: 1.6–52.4%) (MWUT: *p* < 0.001), and correlated positively with the percentages of CD5- (SRCT: *p* = 0.001; r = 0.554), CD8+ (SRCT: *p* = 0.018; r = 0.430), CD16+ (SRCT: *p* < 0.001; r = 0.665) and CD56+ (SRCT: *p* = 0.026; r = 0.406) γδ Tc (Figure 3).

### 3.3. Gamma Delta T Cell Subsets

**Figure 4:**
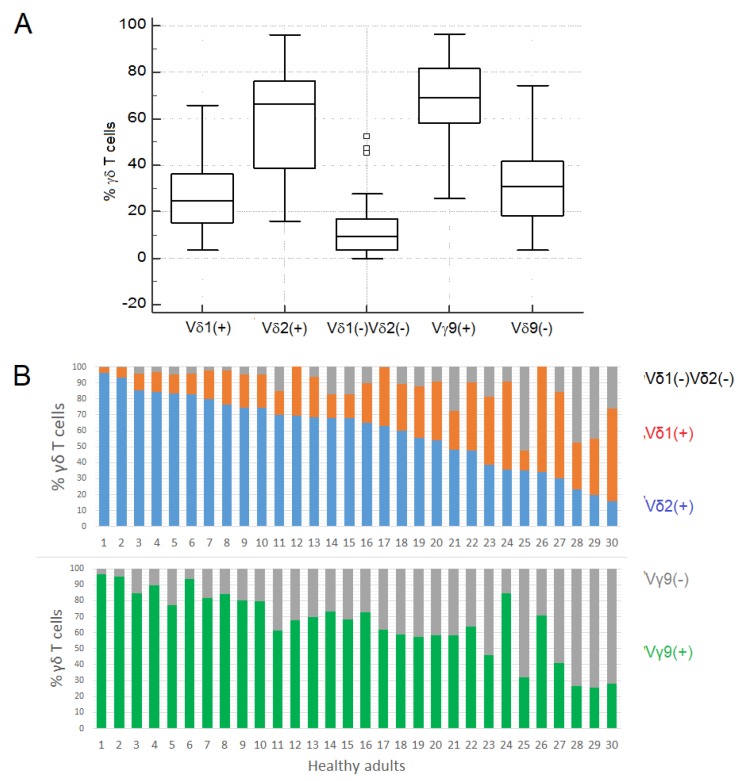
Percentages of Vδ1+, Vδ2+, Vδ1-Vδ2-, Vγ9+ and Vγ9- Tc among peripheral blood γδ Tc in the whole study population (**A**) and in each of the healthy adults studied, numbered by order of decreasing percentage of Vδ2+ Tc, followed by order of decreasing percentage of Vδ1+ Tc (**B**).

As expected, most peripheral blood γδ Tc were Vδ2+ and Vγ9+ (median values: 66.4% and 69.1%, respectively), and most of the remaining γδ Tc were Vδ1+ (median value: 24.6%) (Table 1 and Figure 4A). However, the percentages of Vδ1, Vδ2 and Vγ9 Tc were highly variable, varying from 3.5 to 65.7%, 15.7 to 96.0% and 25.7 to 96.5%, respectively. In one-third of the individuals, the Vγ9Vδ2 subset accounted for <50% of the circulating γδ Tc (Figure 4B).

The median fraction of γδ Tc that were negative for Vδ1 and Vδ2 was 9.2%, ranging from 0.0% to 52.2%, corresponding to cells expressing Vδ chains that were not tested, such as Vδ3 and Vδ5, because the mAbs with these specificities are no longer available in the market (Figure 4). Similarly, the median fraction of Vγ9 negative γδ Tc was 30.9%, ranging from 5.5% to 72.8%, probably corresponding to cells expressing other Vγ regions (e.g., Vγ2, Vγ3, Vγ4, Vγ5, and Vγ8) that were not tested for the same reasons.

**Figure 5:**
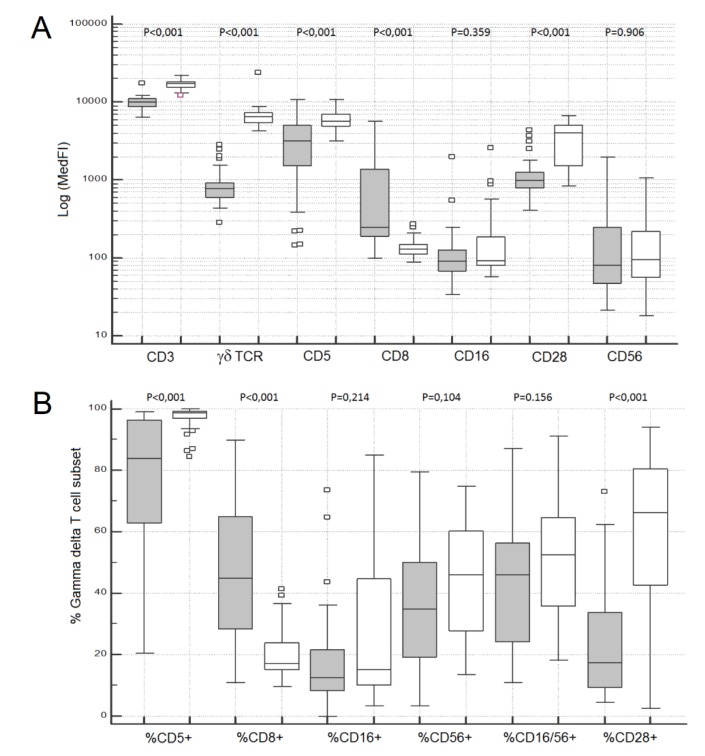
Median fluorescence intensity (**A**) and percentages of positive cells (**B**) observed for CD3, TCR- γδ, CD5, CD8, CD16, CD28 and CD56 molecules in the peripheral blood Vδ1 (gray boxes) and Vδ2 (white boxes) Tc from the study population of healthy adults.

Compared to Vδ2 Tc, Vδ1 Tc had lower levels of CD3, TCR-γδ, CD5 and CD28, and higher levels of CD8 expression, as evaluated by the MedFI (MWUT and WSRT: *p*<0.001 in all cases); no significant differences were observed for the MedFI of CD16 and CD56 expression in total Vδ1 and Vδ2 Tc (MWUT and WSRT: *p* > 0.05 in both cases) (Figure 5A).

Vδ1 Tc also had a higher fraction of CD5- and CD28- cells, compared to Vδ2 Tc (MWUT and WSRT: *p* < 0.001 in both cases), and no significant differences were observed for the percentages of CD16+ and CD56+ cells (MWUT and WSRT: *p* > 0.05 in both cases) (Figure 5B and Table S2). Moreover, in 13 cases (43%), part of the Vδ1, but not of Vδ2 Tc, had high and homogeneous CD8 expression, at levels that approximated those observed in CD8+high αβ Tc.

### 3.4. Age Based Analysis

**Figure 6:**
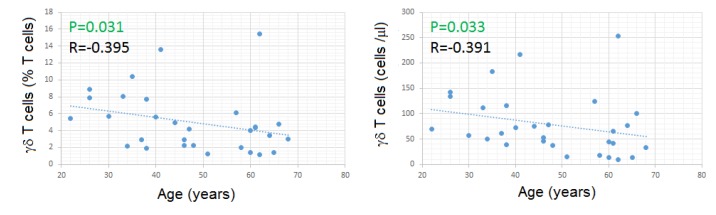
Correlations between age and the percentages and absolute numbers of γδ Tc in the study population of healthy adults. The Spearman’s rank correlation coefficients and *p* values are indicated inside the graphics.

Both the percentages and absolute numbers of γδ Tc had a modest negative correlation with age (Figure 6).

**Figure 7:**
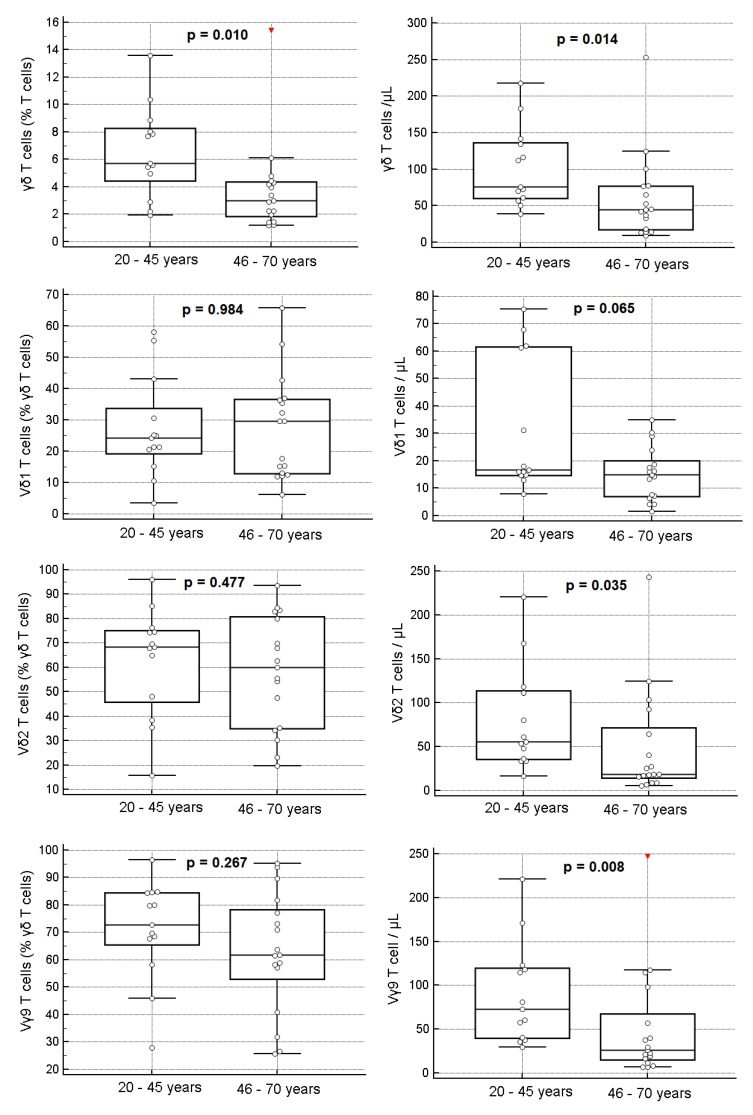
Percentages and absolute numbers of total γδ T cells and γδ T cells expressing Vδ1, Vδ2, and Vγ9, in the peripheral blood from healthy adult individuals 20–45 years old, compared to those 46–70 years old. *p* values (Mann-Whitney U test) are indicated inside the graphics.

In agreement with what has been said above, healthy adults 46 to 70 years old had lower percentages and lower absolute numbers of γδ Tc, compared to those 20 to 45 years old (MWUT: *p* = 0.010 and 0.014, respectively) (Figure 7 and Table S3). In addition, the absolute numbers of all the γδ Tc subsets were lower in older subjects, although reaching significance only for Vγ9Vδ2 Tc (MWUT: *p* = 0.008 for Vγ9 and *p* = 0.035 for Vδ2) (Figure 7 and Table S3). The percentages of Vδ1, Vδ2 and Vγ9 Tc among γδ Tc did not differ significantly between the mentioned age groups (Figure 7 and Table S3).

**Figure 8:**
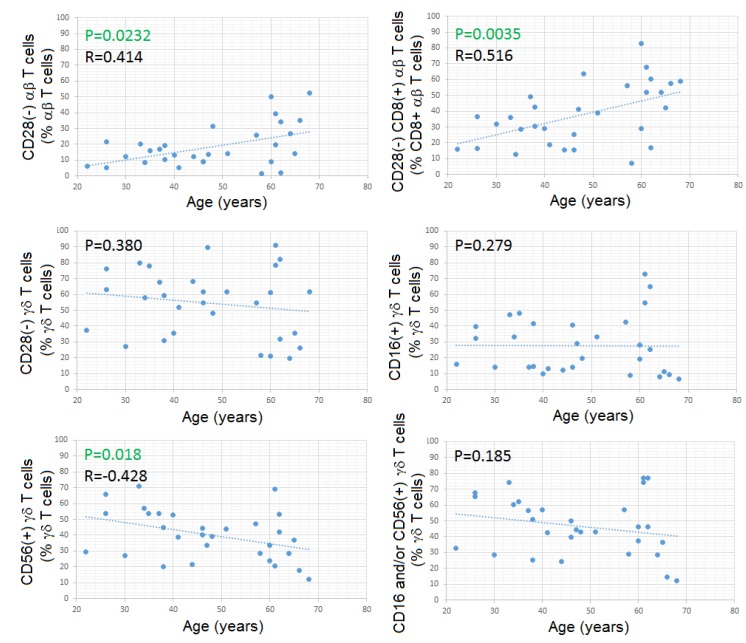
Correlations between age and the percentages and absolute numbers of CD28- αβ Tc, CD8+CD28- αβ Tc, CD28- γδ Tc, CD16+ γδ Tc, CD56+ γδ Tc and CD16/CD56+ γδ Tc in the study population of healthy adults. The Spearman’s rank correlation coefficients and *p* values are indicated inside the graphics.

Contrarily to what happened with CD28- αβ Tc, and especially CD8+high CD28- αβ Tc, both of which increased with aging, we found no significant correlation between the fraction of CD28- γδ Tc and age, nor between CD16+ γδ Tc, and age (Figure 8); unexpectedly, the fraction of CD56+ γδ Tc correlated inversely with age (SRCT: *p* = 0.018; R= -0.427) (Figure 8), this correlation being specific for Vδ2Tc (SRCT: *p* = 0.025; R= –0.408).

### 3.5. Gender-Based Analysis

**Figure 9:**
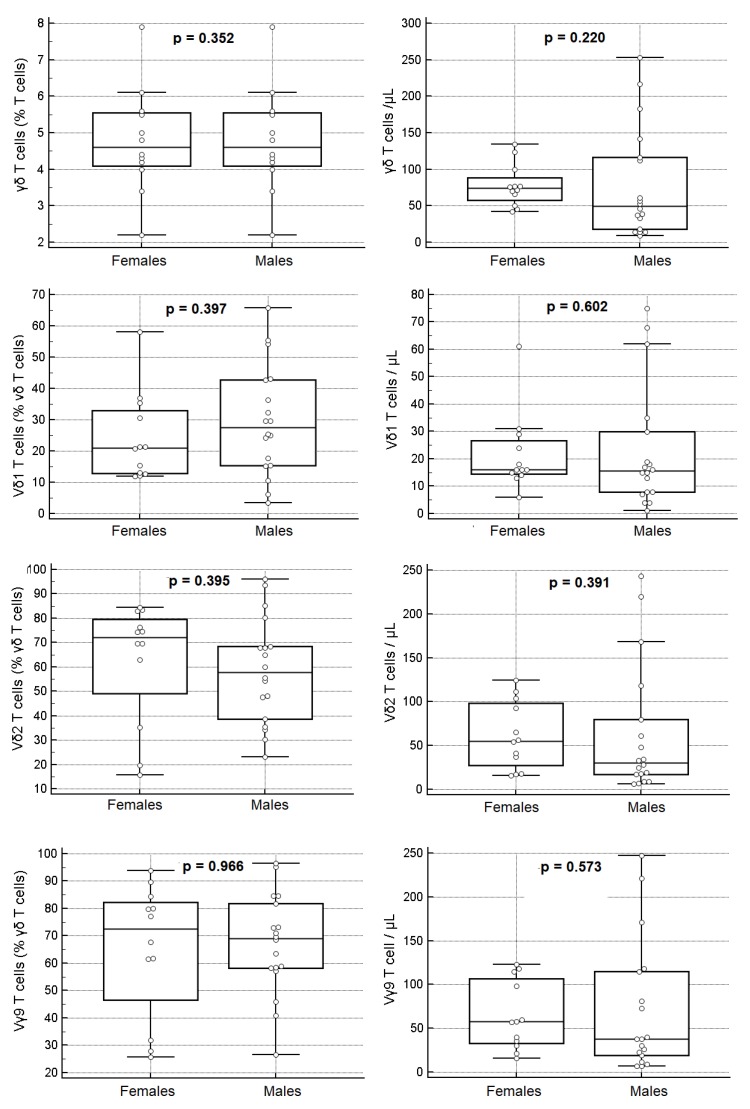
Percentages and absolute numbers of total γδ T cells and γδ T cells expressing Vδ1, Vδ2 and Vγ9, in the peripheral blood of healthy adult females, compared to healthy adult males. *p* values (Mann-Whitney U test) are indicated inside the graphics.

The percentages and absolute numbers of γδ Tc and Vδ1, Vδ2 and Vγ9 Tc subsets showed no significant gender differences when the overall study population was considered (Figure 9 and Table S4) (MWUT: *p* > 0.05 in all cases), although the median values of γδ Tc and Vδ2Vγ9 Tc were lower in males than in females. We also noticed that the percentages of γδ Tc were more variable in males than in females (FT: *p* < 0.001), as were their absolute numbers (FT: *p* = 0.003), due mainly to variations in the Vδ2Vγ9 Tc subset (Figure 9 and Table S4).

### 3.6. Combined Analysis for Age and Gender

**Figure 10:**
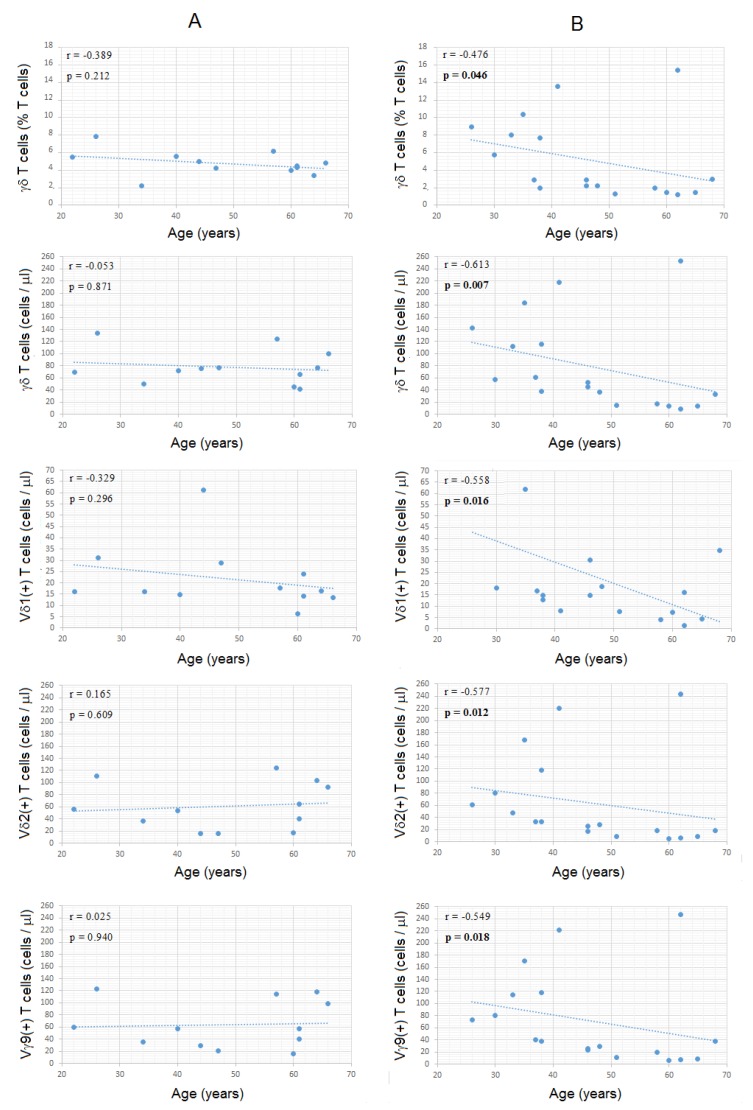
Relations between age and the percentages and absolute numbers of γδ Tc, and the absolute numbers of the γδ Tc subsets in the study population of healthy adult females (**A**) and males (**B**). The Spearman’s rank correlation coefficients and *p* values are indicated inside the graphics.

When age and gender were considered together, we found significant differences between males and females concerning the relation between various immunological parameters and aging, despite the low number of individuals in each group. Indeed, a negative correlation was found for both the percentages and absolute numbers of γδ Tc and age in males (SRCT: *p* = 0.046 and *p* = 0.007, respectively), but not in females (SRCT: *p* > 0.05 in both cases), and similar observations were done for the absolute numbers of Vδ1, Vδ2 and Vγ9 Tc (SRCT males: *p* = 0.016, *p* = 0.012, and *p* = 0.018, respectively; SRCT females: *p* > 0.05 in all cases) (Figure 10, Table S5 and Figure S1). In contrast, the percentages of Vδ1, Vδ2 and Vγ9 Tc subsets among total γδ Tc and the ratio between Vδ2 and Vδ1 Tc did not correlate with age, neither in females nor in males (SRCT: *p* > 0.05) (Figure 10, Table S5 and Figure S1).

Linear regression analysis revealed that circulating γδ Tc drop less strikingly in women than in men, as demonstrated by the slope of the regression equations obtained in females and males (Table S6). Curiously, considering the age groups mentioned above (20–45 and 46–70 years old), there were significant differences between females and males in older but not in younger individuals, with older males having significantly lower percentages and lower counts of γδ Tc (MWUT: *p* = 0.006 and *p* = 0.019, respectively) (Table S7). Older males also had a tendency for having lower counts of Vδ2 and Vγ9 Tc, when compared to older females (MWUT: *p* = 0.064 in both cases) (Table S7). The percentages of Vδ1, Vδ2 and Vγ9 Tc among total γδ Tc and the Vδ1 Tc counts did not differ significantly between the younger and older healthy subjects, either in males or in females (MWUT: *p* > 0.05 in all cases) (Table S7).

### 3.7. Normal Reference Intervals

In an attempt to obtain the normal reference interval for the parameters analyzed, we calculated the 95th ranges using three different methods: 97.5th percentile of the ordering data, assuming a normal distribution; non-parametrical percentile method; and “robust method” also known as percentile bootstrap method, recommended by the CLSI for small samples [60]. Due to the high variation observed, and possibly also because of the small study sample, the lower limits of many parameters had negative values regardless of the method used, and, in some cases, the upper limits of percentage values exceeded 100%, which is also not biologically possible (Table S8).

## 4. Discussion and Literature Review

Despite the enormous progress made over the last decade, the development of γδ Tc is still puzzling, as are their immunophenotypic and functional properties. Biological characteristics such as race, age, gender and past immunological experiences have been shown to affect the relative and absolute numbers of the γδ Tc and their subsets in the PB, and their immunophenotype and function. However, only a few studies were performed in healthy subjects, and it is hardly difficult to compare the results obtained, not only due to the variations in the study populations (e.g., race, age, and gender) but also because of missing data and differences in reporting of the results obtained. The latter comprises graphic representation vs. tabulated data; percentages vs. absolute numbers; and different types of summary statistics, including diverse measures of central tendency (e.g., median and mean) and spread (e.g., standard deviation, range and quartiles). Thus, further comprehensive studies of γδ Tc from healthy persons are needed to better understand the normal immune system and to interpret the data obtained from patients.

Contrasting with the high number of studies investigating the Vγ/Vδ usage in pathological conditions, normal reference intervals for Vδ1, Vδ2 and Vγ9 Tc are not available, and only a few studies aimed to quantify the γδ Tc subsets in healthy adults were performed to date. To the best of our knowledge, our study is one that provides more information about the population evaluated and the variables analyzed, despite the small sample size.

### 4.1. Total γδ T Cells in the PB

There is controversy about expressing the PB lymphocyte populations using relative (%) or absolute cell counts (cells/μL). For instance, absolute CD4+ Tc counts are an important measure of the immune status and are preferred over the percentage of CD4+ Tc in predicting the risk of acquired immunodeficiency syndrome (AIDS)-related illnesses in HIV+ individuals [61]. Absolute cell counts are also considered for the diagnostic criteria of many LPD, as defined by the WHO Classification of Tumors of Hematopoietic and Lymphoid Tissues [62]. However, only a few studies on γδ Tc have expressed the results as absolute cell counts.

The absolute numbers of γδ Tc obtained in our study (median values of 63 γδ Tc/μL, ranging from 9 to 253; 78 ± 60 γδ Tc/μL), and their percentages among total Tc (4.3%, ranging from 1.2% to 15.4%; 5.0 ± 3.6), were similar to those obtained in a series of 157 Spanish healthy adults (78 males median age of 57 years), who had a median percentage of γδ Tc of 3.1% (among total lymphocytes), ranging from 0.2 to 14.0, and a median count of 70 γδ Tc/μL, varying from 5 to 319 [63]. Grossly speaking, similar results were obtained by Michishita et al. in a series of 120 Asian Japanese healthy adults (53 males mean age of 40 years: 68 ± 44 γδ Tc/μL, ranging from 4 to 207) [55].

### 4.2. Gamma-Delta T subsets in the PB

The development of mAbs specific for TCR Vγ and Vδ families allowed for the characterization of the complete TCR-γδ repertoire since the late 80s, although most of the initial studies have analyzed Tc clones derived from PB instead of PB Tc [64,65,66]. Concerning to the Vγ chains, the first studies have identified Vγ9 as the most frequently used Vγ chain in the PB from healthy adult persons, followed by Vγ2/Vγ3. In accordance, it was found that, on average, 80% of the circulating γδ Tc expressed Vγ9, 12% Vγ2/Vγ3, 4% Vγ4, and 8% of one of the remaining Vγ regions (Vγ5, Vγ8, Vγ10 or Vγ11); in addition, 83% of the Vγ9 Tc used Vδ2 and only 13% used Vδ1, whereas the majority (70%) of Vγ2/Vγ3/Vγ4-bearing Tc co-expressed Vδ1 and only 18% co-expressed Vδ2. In overall, the majority (50–90%) of γδ Tc in the PB of healthy adults were shown to use Vγ9 and Vδ2 as variable elements [65].

In studies where TCR-Vγ and Vδ families were expressed as percentages, these were calculated as fractions of total lymphocytes [54,55], total Tc [53] or γδ Tc [23,51,53]. Expressing the results as percentages of total Tc or total lymphocytes may introduce bias related to variations in the percentages of γδ Tc among Tc and the percentages of Tc among lymphocytes, respectively. Thus, similarly to what happens in the studies of TCR-Vβ Tc repertoire, in which the results are usually presented as percentages of cells expressing each of the TCR-Vβ families among the corresponding αβ Tc population (e.g., CD4+ or CD8+ Tc), we consider it to be more suitable to express the results of the Vγ and Vδ T cell repertoires relative to γδ Tc.

In 2006, Sandberg et al. analyzed the expression of Vδ1, Vδ2 and Vγ9 in the PB from 15 healthy Danish adults, and they reported frequencies of γδ Tc expressing the mentioned variable regions ranging from 10% to 15% for Vδ1, 80–85% for Vδ2 and 85–90% for Vγ9 [23]. These ranges are narrower than those obtained in our study (4–66%, 16–96% and 26–97%, respectively). However, in Sandberg’s study, the age and gender of the study population were not mentioned, nor were the median, mean or SD values of the percentages obtained for the analyzed Tc populations.

In the Japanese study of Michishita et al. published in 2011, the average ± SD and minimum-maximum values of blood Vδ1 and Vδ2 Tc were 16 ± 12 (2–52) and 43 ± 36 (1–153) cells/μL [55].

Other relevant papers published more recently—which included two small [67,68] and two large [69,70] studies where healthy individuals were divided in age groups and according to the CMV status—have mainly graphic presentations, and the descriptive summary statistics were not tabulated or presented in the text, impeding the possibility of those being used as reference values; besides, the supplementary material from one of the largest studies is available only for authorized users [69]. These studies will be discussed further below, regarding the effect of age and CMV status on γδ Tc.

### 4.3. Effect of Race/Ethnicity

We found only three small studies reporting on the influence of race/ethnicity on the percentages of γδ Tc and their subsets in the PB [51,54,71].

Esin et al. (1996) investigated the frequency of γδ Tc in the PB of 26 Turkish, 24 Swedish, 35 Asian Japanese and 14 Asian non-Japanese, healthy volunteers, and they found that the median percentages of γδ Tc (among Tc) in the Turkish and Asian non-Japanese were significantly higher than those observed in the Swedish and Asian Japanese groups, with Turkish and Asian non-Japanese having a higher incidence of cases with >10% γδ Tc compared to Swedish and Asian Japanese [71].

Hviid et al. (2000) analyzed the percentages of total γδ Tc and Vδ1 Tc in the PB from 10 Caucasian Danish and eight (West African) Ghanaian healthy adults (age and gender not provided) [51]. They observed that the absolute numbers of total Tc did not differ between groups, but the frequencies of γδ Tc (among total Tc) in West Africans were about twice those of Caucasian Europeans, mainly due to an increased representation of Vδ1 Tc. However, data ware presented mainly graphically, and most descriptive statistic values were not provided [51].

Some years later, Cairo et al. (2010) obtained PB specimens from 32 African American (13 males median age of 47 years) and 33 Caucasian American (14 males median age of 41 years) blood donors [54], and they found significantly higher fractions of Vδ2 Tc in the first group, with mean ± SD percentages of Vδ2 cells in total lymphocytes of 3.7% ± 4.4% and 1.2% ± 2.1%, respectively.

In the Michishita’ series (2011) of Asian Japanese healthy adults mentioned above, the mean percentages of Vδ2 Tc among total lymphocytes were 3.0% ± 3.1% [55]; thus, they were between those observed by Cairo et al. for African American and Caucasian American adults [54]. In our series of Caucasian European adults, the correspondent percentages were 2.4% ± 2.1%.

With respect to absolute cell counts, the values obtained in the Japanese series were 43 ± 36 (1–153) Vδ2 Tc/μL and 16 ± 12 (2–52) Vδ1 Tc/μL [55]. The other series mentioned above did not provide the absolute cell numbers. However, some studies in which the γδ Tc subsets were investigated in pathological conditions have provided the values obtained in healthy subjects used as controls. As an example, Re et al. (2005) evaluated 10 Caucasian, Italian, healthy volunteers (median age of 39 years; gender not available) as controls for patients with melanoma, and they found 83 ± 34 Vδ2 Tc/μL and 31 ± 12 Vδ1 Tc/μL [72]. In addition, Henriques et al. (2016) studied 20 Caucasian, Portuguese, healthy volunteers (mean age of 52 years; 4 males) as controls for patients with Scleroderma, and they found mean counts of Vγ9+Vδ2+ and Vγ9-Vδ2+ of 51±81 and 2 ± 3 Tc/μL, respectively, and 10± 8 Vγ9-Vδ2- Tc/μL, the later corresponding most probably to Vδ1 Tc [73].

Generally speaking, the values obtained in our series of Caucasian, Portuguese, healthy adults, for Vδ2 (63 ± 62; 6–243 Tc/μL), and for Vδ1 (23 ± 19; 2–75 Tc/μL) Tc, were within the series of Caucasian, European, healthy individuals mentioned above, in which the median and range values were not provided.

Differences in γδ Tc between racial and ethnic groups may result from environmental factors and/or genetic influences and needed to be further explored.

### 4.4. Effect of Age

The ontogeny of γδ Tc precedes that of αβ Tc, and γδ TCR gene rearrangements can be detected by embryonic week 8 in humans [74,75,76].

Previous studies have shown that neonatal γδ Tc express diverse Vγ and Vδ regions paired in a variety of combinations rarely observed in the PB from adults, and that Vδ1 Tc predominate over Vδ2 Tc in the CB from neonates [23,38,77,78]. The absolute numbers of peripheral blood γδ Tc were shown to increase from birth to about 10 years of age, due to the expansion of the Vγ9Vδ2 Tc subset, going from a minor population in the CB to the majority of circulating γδ Tc in adults [23,38]. Vγ9Vδ2 Tc expansion in the first decade of life presumably results from the exposure to environmental Ags, such as microbial phosphoantigens and alkylamines [6].

In accordance, in the early 90s, Parker et al. reported that proportion of Tc expressing TCR-γδ in CB was quite similar in all cases tested (mean of 1.7%) and threefold lower than the proportion of Tc expressing TCR-γδ in adult PB (mean of 5.7%); besides, they observed that in the CB from neonates, γδ Tc use predominantly Vδ1 (50% of γδ Tc) and less frequently express Vδ2 (25% of γδ Tc), as compared to values of <30% and >70% in the PB from adults, respectively [38]. They also showed that the proportion of Tc expressing TCR-γδ gradually increased to an average of 10% at 6 years of age, after which it fell to a lower level in adulthood [38].

More recently, Sandberg et al. (2006) described a fivefold increase of absolute numbers of γδ Tc during the first 2 years of life, followed by a gradual decrease to adult levels [23]. They also compared the expression of Vγ9, Vδ1 and Vδ2 on γδ Tc in the CB from neonates (*n* = 10) and in PB samples from healthy children (*n* = 15) and healthy adults (*n* = 15), and they found that in neonatal CB, approximately half of the γδ Tc expressed Vδ1 (as compared to 10–15% in adults), while 30–40% used Vγ9 and/or Vδ2 (as compared to Vδ2: 80–85% and Vγ9: 85–90% in adults) [23].

Little was known about the relative representation of the γδ Tc subsets in the human PB before birth until 2015, when Dimova et al. reported on the Vγ and Vδ usage in the PB from human fetuses (*n* = 87) at different gestation times [56]. Unexpectedly, they observed that Vγ9Vδ2 Tc were the predominant γδ Tc subset in the PB of the second-trimester fetus (75–80% of the γδ Tc), i.e., before postnatal microbial exposure, and that the second most abundant population was Vγ9Vδ2+ (15–20% of γδ Tc), whereas Vδ1 and Vδ3 Tc were present only at low frequencies [56]. At later gestational times, Vδ2 Tc in fetal PB decreased significantly, while Vδ1 Tc increased continuously, representing the major γδ Tc subpopulation in the PB at term delivery (15–20% of γδ Tc). They also showed that fetal Vγ9Vδ2 Tc were responsive to phosphoantigens and had limited diversity in the CDR3 of the Vγ9 chain gene, with a germline-encoded sequence accounting for >50% of all sequences, in association with a prototypic complementary determining region 3 (CDR3)δ2; furthermore, fetal blood Vγ9Vδ2 Tc were preprogrammed for an effector T helper (Th) type 1 (Th1) phenotype, with properties of rapidly activatable Tc [56].

Major age-related changes in the γδ Tc compartment have also been reported in healthy adults, and they were shown to be more accentuated in old people and centenarians. In line with that, Argentati et al. (2002), in a series of 104 Italian healthy volunteers, observed that the absolute numbers of circulating γδ Tc were significantly less prevalent in centenarians (mean age: 100 years,) and old people (mean age: 80 years) compared to young adults (mean age: 34 ± 6 years), as a consequence of the age-related decrease in lymphocyte numbers [52]. They also observed that the decrease of γδ Tc counts with aging resulted from a decrease of Vδ2 Tc (mean counts of 77, 31 and 23 cells/μL, for young and old adults and centenarians, respectively), whereas the absolute number of Vδ1 Tc was unaffected by age (mean counts of 41, 34 and 38 cells/μL, respectively); consequently, the Vδ2/Vδ1 ratio was inverted in the PB from old subjects and centenarians [52].

Subsequently, Re et al. (2005) also found that upon activation *in vitro*, the percentage of tumor necrosis factor-alpha (TNF-α) producing γδ Tc was higher in old in comparison with young/adult healthy subjects, whereas no significant differences were noted in the percentage of γδ Tc producing interferon-gamma (IFN-γ) [79]. Thus, they demonstrated that γδ Tc from older individuals were not only reduced in number, they were also functionally different. In the same year, Re et al. realized that the decreased absolute numbers of Vγ9Vδ2 Tc in old individuals in comparison with young and middle aged adults resulted from a reduction of naïve (T_N_) and central memory (T_CM_) Vδ2 Tc, bearing CD27 and CCR7 receptors, whereas the proportion of effector memory (T_EM_) Vδ2 Tc, lacking CD27 and CCR7, was significantly increased [72]. Subsequent functional studies revealed that T_EM_ Vδ2 Tc were increased after in vitro culture in the presence of IPP and interleukin (IL)-2, in contrast to that observed in old subjects, thereby confirming a lack of T_N_ and T_CM_ responding to IL-2. Finally, they observed that perforin-containing, terminally differentiated effector (T_EMRA_) Vδ2 Tc showed no age-related differences. These data demonstrated a shift of the circulating γδ Tc towards T_EM_ and T_EMRA_ in the elderly, with reduction of T_N_ and T_CM_ cells [72].

One year later, Caccamo et al. (2006) studied 320 Italian healthy subjects aged from 2 to 70 years-old, and they observed that the percentage of peripheral blood Vγ9Vδ2 Tc (in total Tc) raised from birth to puberty, and gradually decreased beyond the age of 20–30 years [53]; they analyzed separately, three age groups (2–15, 20–30 and 30–60 years-old), and they found that the percentages of Vγ9Vδ2 Tc (among total Tc) decreased progressively in children, younger adults and older adults (mean values of 3.5%, 3.0% and 1.5%) [53]. Furthermore, they demonstrated that loss of peripheral Vγ9Vδ2 Tc with aging was accompanied by changes in the distribution of functional Tc subsets. By splitting Vγ9Vδ2 Tc into T_N_ (CD27+CD45RA+), T_CM_ (CD27+CD45RA-), T_EM_ (CD27-CD45RA-) and T_EMRA_ (CD27-CD45RA+), they observed a progressive decrease in T_EM_ and T_EMRA_ in the Vγ9Vδ2 Tc compartment with aging, with a concomitant enrichment in T_CM_. The former proliferated more poorly than the later in response to stimulation with IPP in vitro but produced higher amounts of IFN-γ [53]. Once again, most of the values were only represented graphically.

A few years later (2011), Michishita et al., when examining the γδ Tc repertoire in the series of 120 Asian Japanese healthy volunteers mentioned above, also observed that the absolute numbers of γδ Tc decrease with aging, as the result of reduction of Vδ2, but not of Vδ1 Tc [55]. To understand why Vδ2 Tc had an age-dependent decrease, they studied the expansion capability of Vδ1 and Vδ2 Tc after culture with phytohemagglutinin (PHA), in the presence or absence of IL-2. They observed that, in contrast to Vδ1 Tc, Vδ2 Tc rapidly died after stimulation with PHA, and that this phenomenon was not rescued by adding IL-2. They also found that the fraction of apoptotic cells was higher among Vδ2 Tc and that Bcl-2 expression was down-regulated in stimulated Vδ1 Tc, but not in stimulated Vδ2 Tc. They concluded that age-related decrease of Vδ2 Tc may be explained, at least in part, by a higher susceptibility to activation-induced cell death [55].

Cairo et al. (2010) also described that the percentages of Vδ2 Tc (among total lymphocytes) declined as age increased, and that rates of decline were the same for Caucasian Americans and African Americans [54], and Hviid et al. (2000) confirmed that the frequencies of Vδ1 Tc (among γδ Tc) did not depend on age [51].

In a study published by Roux et al. in 2013, in which 70 healthy adults were divided into age groups and according to the CMV serological status, a decrease in the frequency of γδ Tc was also observed with aging; however, the frequency of Vδ2 Tc decreased substantially with aging regardless of CMV status, while the percentage of Vδ2-negative Tc decreased mainly in CMV-seronegative subjects (please see below) [67].

In the same year, Wistuba-Hamprecht et al. reported on the percentage and immunophenotype of peripheral blood Vδ2 Tc from 33 healthy donors (mean age: 52 years; 12 males) [68], and two years later, they published a large study that included 217 participants (23 to 85 years, gender not provided) of the Berlin Aging Study II (BASEII) study [69]. In both studies, the participants were aggrouped accordingly to age and CMV-serostatus, and Vδ1 and Vδ2 Tc were evaluated. In the first study, Wistuba-Hamprecht et al. observed a trend towards age-associated reduction in the frequency of Vδ2 Tc when CMV-seropositive and seronegative subjects were poled together, and significant accumulation of Vδ2-negative Tc with a late-differentiated (CD27-CD28-) phenotype with aging, the latter occurring only in CMV-seropositive subjects [68]. The second and largest study confirmed these observations, with older subjects having lower Vδ2 Tc counts regardless of the CMV serostatus; stratifying subjects according to age did not reveal any significant differences in Vδ2 Tc, either for early (CD27+CD28+CD45RA+CD16-) or for late-differentiated (CD27-CD28-CD45RA+CD16-) γδ Tc [69]. Changes observed in Vδ1 depended mainly on the CMV serostatus and are described below.

More recently, other large study (157 healthy adults, 20–95 years-old, 58 males) published by Kallemeijn et al. in 2017, also aimed to evaluate the impact of aging and latent CMV infection on the immunophenotypic features of γδ Tc, including those expressing the TCR-Vγ9, Vγδ1 and Vδ2 families [70]. They also found a significant decrease in total γδ Tc numbers with aging, which was paralleled by a significant decrease of Vδ2 Tc but not of Vδ1 Tc [70]. In addition, they described a decrease in absolute numbers of effector memory (CD45RO+CD197-) and effector (CD45RO-CD197-) γδ Tc, without significant differences in numbers of naïve (CD45RO-CD197+) and central memory (CD45RO+CD197+); expressing the results as percentages, there was a decrease in naïve and an increase in effector γδ Tc, which were already visible from age 50 onwards—more pronounced in the oldest age group (>70 years) [70]. These changes were accompanied by a shift from less differentiated (i.e., CD27-/+CD28+) to a more differentiated (i.e., CD27-CD28-) phenotype, especially in the oldest age group, although there was a decrease in absolute cell numbers of all these γδ Tc populations [70].

In agreement to the studies mentioned above, we observed that in Caucasian, Portuguese, healthy adults aged from 20 to 70 years the peripheral blood γδ Tc decreased with aging [52,55,67,80], and that the relative representation of the Vδ1 and Vδ2 subsets among γδ Tc did not vary significantly. Additionally, in line with these studies [52,55], we found that the decrease of the absolute numbers of γδ Tc with aging resulted mainly from a reduction of Vγ9Vδ2 Tc, with less relevant changes in the Vδ1 Tc compartment. It is worth noting that our series did not include the extremes of life, where major changes in the γδ Tc repertoire are expected to occur.

### 4.5. Effect of Gender

When studying the series of Italian blood donors mentioned above (*n* = 224, 20–60 years, 111 males), Caccamo et al. were the first to demonstrate gender bias in γδ Tc [53]. They realized that the percentage of Vδ2 Tc (among Tc) did not differ between males and females under 15-year-old, but they were significantly less prevalent in males than in females aged from 20–30 years, and especially, in those aged from 30–60 years. They also found that the depletion of T_EM_ and T_EMRA_ was more pronounced in adult males than in adult females [53]. Thus, it seems that age-related differences in Vγ9Vδ2 Tc between males and females only manifest after puberty, probably because of hormonal or other physiological factors.

In contrast, Cairo et al. did not find significant differences between Italian healthy males and females (*n* = 65, 19–64 years, 38 males), concerning the percentage of Vδ2 Tc among total lymphocytes [54], and Michishita *et al*. realized that the numbers of γδ Tc and Vδ2 Tc were significantly higher in 42 Japanese healthy adult males, as compared to Japanese healthy adult age-matched females [55].

Concerning the impact of gender on the analyzed Tc populations, we feel that in our study, as in the Cairo’s study [54], larger sample sizes could have revealed an influence of gender similar to that found by Caccamo *et al*., who reported higher Vδ2 Tc counts in women compared to age-matched men [53]. We have no obvious explanation for the opposite results obtained by Michishita et al. [55].

Similarly to Caccamo et al. [53], we observed that the percentage of Vδ2 Tc dropped later and more slowly in females than in males, and although we did not obtain significant differences when considering the overall study population, we found that middle aged females had significantly higher levels of Vδ2 Tc than middle aged males. Thus, circulating γδ Tc remain elevated for a longer period in life in women, dropping slowly than in men, an effect that is mostly observed in Vγ9Vδ2 Tc [53].

### 4.6. Effect of Pregnancy

Previous studies have revealed significant changes in circulating γδ Tc during pregnancy, peri and postpartum, as part of the physiological changes of adjustment of the immune system, and that uterine decidua γδ Tc play an important role in fetal-maternal relationship, a process that seems to be hormone-dependent [81,82,83].

In accordance, in the uterine decidua from pregnant women, γδ Tc significantly increase in number, and most of them are activated Vδ1 Tc, which recognize conserved sequences on the trophoblast [84]. In the late 90s, it was reported that the percentage of γδ Tc in the PB from healthy pregnant women is significantly higher, with a lower fraction of Vγ9Vδ2 and a higher fraction of Vγ1.4 (now designated as Vγ4) Vδ1 Tc, compared to women with recurrent miscarriages and healthy non-pregnant women [85,86]. Signaling via the Vγ4Vδ1 was shown to induce Th2 type polarization, whereas Tc activation via Vγ9Vδ2 resulted in a Th1 type response [86]. More recently, it was shown that the initiation of labor is characterized by changes in the V-chain usage of γδ Tc, with increased percentages of Vγ9Vδ2 and decreased percentages of Vγ4Vδ1 Tc [87].

Further studies on the γδ Tc repertoires in specific periods of women life, such as on the menarche and menstrual cycles, will probably contribute to elucidating the physiological role of γδ Tc.

The results obtained in our study were not influenced by pregnancy, as women are deferred from blood donation during pregnancy, and for 6 months after delivery or abortion.

### 4.7. Pathological Conditions Involving γδ T Cells

Increased relative and/or absolute numbers of γδ Tc have been described in association with several pathological conditions. Granel et al. (2002) reviewed the clinical data of 55 patients with γδ Tc lymphocytosis, defined by a proportion of γδ Tc over 10% of the peripheral blood Tc, and they observed three groups of pathologies: infectious (e.g., viral infections and tuberculosis), inflammatory (e.g., sarcoidosis and autoimmune diseases) and hematological (e.g., monoclonal gammopathy) [2].

When investigating the diseases associated with an increase of γδ Tc in the PB, in patients observed at the Mayo Clinic, Roden et al. (2008) considered arbitrarily γδ Tc to be increased when they comprised more than 5% of PB lymphocytes and/or when their absolute number exceeded 200 cells/μL [3]. They noticed that 62 (18%) of 352 PB samples analyzed consecutively had an increase in γδ Tc. In 28 out of 36 cases (78%) where clinical information was available, there were one or more associated conditions, consisting of infectious/inflammatory pathologies, autoimmune diseases, lymphoproliferative disorders and previous splenectomy [3].

Among the pathological conditions that have been associated with a γδ Tc-mediated immune response, only infections by human herpes virus (HHV) will be discussed in detail, because of their ability to infect and to persist in the host without causing clinical manifestations. Detailed analysis of the role of γδ Tc in other infections, and in autoimmune and neoplastic diseases, is outside the scope of this work, since all individuals examined were healthy.

#### 4.7.1. Viral Infections

##### Herpesviruses

Most of the herpesviruses known to cause diseases in humans are ubiquitous viruses that share the ability to remain latent within the body, to interact with immune cells in order to create a permissive environment and to escape immune surveillance, reactivating in immunodepressed patients; some of them have also been implicated in the genesis of malignant neoplasms [88]. Of the HHVs, only cytomegalovirus (CMV, HHV-5) has been exhaustively investigated, concerning both the participation of γδ Tc in the immune response against the virus, and its ability to influence the γδ Tc repertoire.

##### Cytomegalovirus/Human Herpes Virus Type 5

Human CMV (HHV-5) usually infects the host during childhood, establishing life-long latency [89]. The CMV genome contains several accessory genes, most of which are involved in immune evasion, resulting in a symbiosis between virus and host [90]. However, the mechanisms by which CMV maintains latency and reactivates in immunocompromised subjects are not completely understood. In immunodepressed patients, CMV infection can be life-threatening, causing organ injury (e.g., hepatitis, pneumonitis, retinitis, meningitis and encephalitis), and systemic disease; intrauterine CMV infection may cause microcephaly, sensorineural hearing loss and mental retardation [91,92].

The first evidence of involvement of γδ Tc in the immune response against CMV dates to 1999, when Déchanet et al. detected an expansion of γδ Tc in 31 of 205 renal allograft recipients and identified CMV infection as the only independent variable associated with high levels of circulating γδ Tc [93]. In the same year, they showed that Vδ1 and Vδ3 Tc were preferentially expanded in these patients, and they found a marked restriction of CDR3 size distribution in Vδ3, and to a lesser extent, in Vδ1 Tc; furthermore, they observed that Vδ1 and Vδ3 Tc from CMV-infected kidney recipients were able to proliferate in vitro in response to CMV [94]. Subsequent reports have confirmed that γδ Tc are implicated in the control of CMV infection, which is mediated through Vδ2-negative, mainly Vδ1+ or Vδ3+, but also Vδ5+ Tc [95,96].

In healthy subjects, seropositivity for CMV has been related with higher frequencies and absolute numbers of Vδ2-negative Tc in the PB with a restricted repertoire and a differentiated immunophenotype [67,68,69,70,97]. It seems that aging and CMV infection independently impact γδ Tc, providing an explanation for different changes observed in Vδ2-negative and Vδ2+ Tc in the elderly [67].

In conformity to what was said above, Pitard et al. (2008) observed that the percentages of Vδ2-negative γδ Tc were increased in CMV-seropositive compared with -seronegative healthy subjects [97]. Additionally, in seropositive subjects, Vδ2-negative γδ Tc exhibited a more differentiated cytotoxic effector/memory phenotype (CD45RA+ CD27− CD28− CD62L-), which was also observed in transplanted patients challenged with CMV, whereas in seronegative people Vδ2-negative γδ Tc had mainly a naïve (CD45RA+ CD27+ CD28−/+ CD62L+) phenotype. Moreover, Vδ1 Tc from CMV-seropositive donors showed a more restricted repertoire than those from seronegative ones, as revealed by CDR3 size distributions of the TCR Vδ1 sequences. They concluded that increased effector-memory Vδ2-negative γδ Tc in the PB traduces an adaptive immune response to CMV infection in both immunocompetent and immunosuppressed individuals [97].

Some years later, Roux et al. (2013) showed that the frequency of Vδ2 Tc declines in with advanced age irrespective of the CMV status, whereas Vδ2-negative γδ Tc only decrease in CMV-seronegative individuals, being preserved overtime in CMV-seropositive subjects, probably because of persistent γδ Tc stimulation [67]. The stimulatory effect of CMV on Vδ2-negative γδ Tc was documented by the expression of activation related markers (e.g., CD38 and HLA-DR) in patients with acute and chronic CMV infection, and using in vitro experiments [67].

In the same year, Wistuba-Hamprecht et al. reported that in CMV-seropositive healthy subjects there was a significantly lower ratio of Vδ2+/Vδ2-negative γδ Tc in older individuals, and an age-associated trend towards a reduction of early-differentiated CD27+CD28+ Vδ2-negative Tc, with concomitant increase of terminally differentiated CD27-CD28- Vδ2-negative Tc, whereas the phenotype of Vδ2 Tc was not affected by a latent CMV infection [68]. These differences were, however, absent in CMV-seronegative donors, in whom they observed a significant reduction of Vδ2-negative Tc.

When studying a large number of individuals from the Berlin Aging Study II, Wistuba-Hamprecht et al. (2015) also found higher percentages of Vδ1 Tc and less abundance of Vδ2 Tc in CMV-seropositive elderly people, resulting in the highest values of Vδ1:Vδ2 [69]. In addition, they observed higher frequencies of late-differentiated (CD27-CD28-) and lower frequencies of early-differentiated (CD27+CD28+) cells among Vδ1+ and Vδ1/Vδ2 negative γδ Tc, but not among Vδ2+ Tc from elderly CMV-seropositive individuals, confirming the association of Vδ2-negative γδ Tc with CMV-immunosurveillance [69].

Finally, the large study performed by Kallemeijn et al. (2017) also revealed a major impact of past CMV infection on γδ Tc, with a shift from naïve to more differentiated effector γδ Tc phenotypes [70]. The main findings observed in Vδ1 Tc were lower proportions of early differentiated (CD27+CD28+CD45RA+) and higher proportions of late-differentiated (CD27-CD28-CD45RA+) cells in young and old CMV-seropositive individuals, when compared to CMV-seronegative individuals of the same age group. Similar findings were found in Vδ1/Vδ2 negative Tc, but not in Vδ2+ Tc [70].

Thus, CMV infection has been considered one of the most important factors for driving immune senescence, having impacts on both αβ and γδ Tc [98,99]. Taking into account that the rates of CMV infection are diverse in different parts of the world, it may also account for race/ethnicity related differences.

##### Other Herpesviruses

Gamma delta Tc have also been implicated in the immune response against other HHV, including herpes simplex viruses type 1 and 2 (HSV-1 and 2), varicella zoster virus (VZV, HHV-3), Epstein Barr virus (EBV, HHV-4),and Kaposi’s sarcoma-associated herpes virus (KSHV, HHV-8), although the evidence is much lower than in CMV (HHV-5).

Herpes simplex viruses types 1 and 2: Only a few studies in animal models have implicated γδ Tc in the immune response against HSV-1 [100,101] and HSV-2 [102,103,104], and studies in humans are even scarcer [105,106]. In humans, HSV-specific cytotoxic activity is mediated mainly by γδ Tc in some persons, whereas in other, αβ Tc are primarily involved [105]. One study revealed that Tc recovered from the lesions of patients with genital herpes and expanded by mitogen stimulation, gave rise to γδ Tc clones that were predominantly Vγ9Vδ2, showed reactivity to IPP and were able to secrete IFN-γ, TNF-α, IL-8, MIP-1α (macrophage inflammatory protein-1 alpha) and RANTES (regulated on activation, normally T cell expressed or secreted), suggesting that IPP-reactive Th1-pollarized Vγ9Vδ2 Tc are involved in the immune response against HSV-2 [106].

Varicella zoster virus—human herpes virus type 3: Evidence for the implication of γδ Tc in the immune response against VZV is also scarce. We found only one study indicating that patients with recurrent aphthous ulceration (RAU), presumably related to VZV reactivation [107], have increased percentages of γδ Tc in the PB [107]. The study population consisted of 13 patients with active RAU, 14 patients with inactive RAU and 18 healthy volunteers without RAU. The mean percentage of circulating γδ Tc (among total Tc) was increased in patients with active RAU, as compared with patients with inactive RAU, and with controls [108].

Epstein Barr virus—human herpes virus type 4: Several studies have indicated that γδ Tc, and particularly the Vδ1 subset, are able to respond to ligands expressed in EBV-infected B cells [109,110,111,112]. Gamma delta Tc may be themselves infected by EBV and then expand clonally, such as occurring in patients with chronic active EBV disease [113,114]. In addition, EBV has been implicated in the genesis of γδ Tc lymphomas, which are mainly extranodal cytotoxic lymphomas [115,116]. Some studies, however, have associated Vγ9Vδ2 Tc to the immune response against EBV, particularly Vγ9-JγP/Vδ2-Jδ3+ γδ Tc, which were shown to be preferentially expanded in patients with nasal γδ Tc lymphoma and chronic active EBV infection [117]. Finally, targeted activation of human Vγ9Vδ2 Tc controls EBV-induced B cell LPD [118].

Human herpes viruses types 6 and 7: Some studies have revealed that when γδ Tc purified from the PB of healthy adults and activated in vitro with PHA, were exposed to HHV-6, they became infected by the virus; in contrast, no signs of infection or were detected after exposure to HHV-7 [119]. Additionally, γδ Tc displayed cytolytic activity against autologous and heterologous target cells infected with HHV-6 [119].

Kaposi’s sarcoma-associated herpes virus—human herpes virus type 8: Barcy et al. found that HHV-8 infection was associated with significant expansion of γδ Tc in the PB, consisting mainly of terminal effector Vδ1 Tc [120]. They showed that in vitro stimulation of PB mononuclear cells from HHV-8-infected subjects with either HHV-8 or viral proteins resulted in Vδ1 Tc activation; in addition, Vδ1 Tc displayed a strong reactivity against HHV-8-infected cell lines and prevented the release of infectious viral particles following lytic replication, suggesting that γδ Tc play a role in the immune response against HHV-8 [120].

The number of studies that have investigated the role of HHV other than CMV in modeling the γδ Tc repertoire in healthy humans is even more limited, and we found only one study addressing this question [55]. In their study of 120 Japanese healthy individuals mentioned above, Michishita et al. investigated whether past viral infections with CMV, EBV, HSV, VZV and Human Parvovirus B19 (HPVB19), affected the γδ Tc repertoire. Confirming previous observations, they found that the numbers Vδ1 Tc (but not Vδ2 Tc) were significantly higher in CMV-seropositive than in CMV-seronegative subjects, and they realized that serological status for HSV and HPVB19 infection did not have impact in Vδ1 and Vδ2 Tc counts; however, they were not able to conclude about the effect of VZV and EVB, because most of the subjects examined had positive serological tests for these viruses [55].

Serological tests for HHV are not included in the recommended tests for blood donors in Portugal, and so, they were not performed in our study population; seroprevalence studies for most of these viruses in the general Portuguese population are also not available. However, it can be grossly estimated using studies performed in other countries with similar characteristics. Taking in account the available data, we estimate a seroprevalence (IgG+/IgM-) >70–80% for HHV-1 (HSV-1), >10–20% for HHV-2 (HSV-2), >90–95% for HHV-3 (VZV), >80–90% for HHV-4 (EBV), >70–80% for HHV-5 (CMV) and >80–90% for HHV-6 and HHV-7.

##### Other Viruses

Several studies have reported on the role of γδ Tc in the immune response against the HIV [9,10], and some studies have also implicated these cells in hepatitis caused by HBV and HCV [11,121]. In addition, HTLV-I was shown to infect γδ Tc, but, to the best of our knowledge, the role of γδ Tc in the immune response to HTLV has not been investigated [122]. All blood donors included in our study have been tested (and were negative) for HIV-1 and 2, HTLV-I and II, HBV and HCV.

#### 4.7.2. Bacterial Infections

Gamma delta Tc, as other “innate” Tc, are known to have an important role in bacterial infections, acting as a bridge between the innate and adaptive immune system [123]. Increased proportions and/or absolute numbers of circulating γδ Tc, mostly of the Vγ9Vδ2 Tc subset, have been observed in various human intracellular bacterial infections—most of the studies having been performed in the 90s [13]. These include tuberculosis [124,125], salmonellosis [126], brucellosis [127], legionellosis [128], listeriosis [129], tularemia [130] and ehrlichiosis [131]. None of the individuals analyzed in our study had clinical evidence of having bacterial infections.

#### 4.7.3. Parasitic Protozoan Infections

Increased proportion and/or absolute numbers of γδ Tc have also been described in parasitic protozoan infections, such as in malaria [132,133,134], toxoplasmosis [135,136] and leishmaniosis [137]. The healthy subjects that participated in our study had no clinical evidence of being infected by parasitic protozoan.

#### 4.7.4. Autoimmune/Inflammatory Diseases

Gamma delta Tc have also been implicated in autoimmune/inflammatory disorders, especially in those that are organ-specific, such as rheumatoid arthritis, autoimmune thyroiditis, autoimmune hepatitis, autoimmune myositis, inflammatory bowel diseases and multiple sclerosis, but also in systemic lupus erythematosus, Sjögren Syndrome, Psoriasis and other systemic diseases [18,19]. None of the individuals analyzed in this study had autoimmune diseases.

#### 4.7.5. Neoplastic Diseases

Alpha/beta and γδ Tc make distinct contributions to anticancer surveillance, as they have distinct activation requirements [15,16,17]. Down-regulation of expression of MHC-class I molecules, and tumor-specific Ags, is observed frequently during tumor progression, resulting in an impairment of MHC-restricted, αβ Tc-mediated tumor-specific immunity. Given the lack of requirement for classical MHC-presenting molecules, γδ Tc may, therefore, represent a potent alternative in anti-cancer surveillance. In addition, subsets of human γδ Tc can identify tumor-expressed ligands that are not seen by conventional αβ Tc. For instance, Vγ1 Tc recognize MIC-A/B and ULBP expressed on epithelial tumors and some lymphoma cells and Vδ2 Tc are activated by phosphoantigens overproduced by tumor cells [5,138]. In accordance, Vδ2 Tc were shown to be capable of killing myeloma and lymphoma B cells by recognizing phosphoantigens produced by these cells [139]; the Vδ1 Tc have been implied mainly in the defense against epithelial cancers, being able to recognize colorectal, renal and pancreatic cancer cells, releasing IFN-γ and exhibiting cytolytic properties [140]. Nevertheless, this dichotomy is not absolute [141,142].

Altered percentages and/or absolute numbers of γδ Tc have been described in the PB from patients with diverse types of hematological [143,144,145] and non-hematological tumors [79,146,147,148,149,150]. These alterations are difficult to systematize and to interpret due to the tumor diversity, disease stage, therapy-related effects and the complexity of the host-tumor interactions. People who had or who have had cancer are impeded of giving blood. Thus, although the possibility of clinical silent not yet identified neoplasm cannot be excluded, it is unlikely that the γδ TCR repertoire described herein was influenced by the existence of malignant neoplasms.

The role of γδ T-cells in the anticancer therapy, is being explored as a novel immunotherapy in many types of cancer [21]. However, recent studies have revealed that in certain conditions γδ Tc may display tumor-promoting functions, and these properties have been linked to IL-17 production [151,152]. Moreover, regulatory γδ Tc in the tumor microenvironment may exert suppressive effects, being that they are associated with poor prognosis [152,153,154,155,156].

#### 4.7.6. Food and Medications

Human Vγ9Vδ2 Tc monitor isoprenoid metabolism by recognizing HMB-PP, an intermediate in non-mevalonate pathway used by microbes, and IPP, an intermediate in the mevalonate pathway used by humans. Bisphosphonates, especially nitrogen-containing bisphosphonates (NBP), such as pamidronate, alendronate and zoledronate, which are widely used to treat osteoporosis, and alkylamines, are derived from microbes and certain edible plants and fruits, such as tea, wine, apples and mushrooms, indirectly stimulate Vγ9Vδ2 Tc by inhibiting farnesyl diphosphate synthase (FDPS) in the mevalonate pathway, thereby increasing the levels of IPP [157,158,159]. In contrast, pharmacological agents that block the mevalonate pathway upstream by inhibiting the enzyme 3-hydroxy-3-methylglutaryl-coenzyme A (HMG-CoA) reductase, such as statins, which are used as anti-lipemic drugs, lead to decreased IPP levels [160,161]. Taking in consideration the variety of bioactive food components and pharmacological agents that can influence γδ Tc activation and function, the impact of the diet and medications on γδ Tc are not simple to interpret. The possibility that the γδ Tc may be altered by medications that do not contraindicate blood donation, such as statins as bisphosphonates, cannot be ruled out in the present study.

### 4.8. Immunophenotypic and Functional Features of Peripheral Blood γδ T Cells

Gama delta Tc are a heterogeneous cell population that combines rapid innate-like with conventional adaptive properties, making essential contributions to the immune response [162].

As for αβ Tc, there are many factors influencing the immunophenotypic features of γδ Tc. Upon stimulation, naïve γδ Tc can sequentially differentiate into T_CM_, T_EM_ and T_EMRA_ cells, thereby modifying the levels of CD45RA, CD45RO, CD27, CD28, CD197/CCR7 and other cell surface receptors [72,163,164]. In addition, once activated, γδ Tc modulate a wide range of cell surface activation-related molecules (e.g., CD69, CD25, CD28, CD38, CD45RO and HLA-DR) [165,166]. Activated γδ Tc were shown to have properties of Ag presenting cells (APC), thereby providing co-stimulatory signals enough for inducting naïve αβ Tc proliferation and differentiation [167,168], and promoting dendritic cell maturation [169].

Depending on the microenvironment (i.e., cytokine milieu) human γδ Tc polarize into Th1 (e.g., IFN-γ-producing), Th2 (e.g., IL-4-producing) and Th17 (e.g., IL-17-producing) cells, making different types of cytokines [170,171], and eventually converting into cytotoxic T lymphocytes (CTL), so playing a role in anti-viral and anti-tumor immune responses. Cytotoxic γδ Tc do express receptors involved in the recognition and/or adhesion to their targets (e.g., CD16, CD56 and different types of killer cell receptors) [172,173], and cytotoxic granules containing granzymes and perforin [174,175]. The chemokine receptor CXCR5 (CD185) identifies a unique subset of Vγ9Vδ2 Tc that expresses the costimulatory molecules CD278 (ICOS, inducible T-cell costimulatory molecule), CD279 (programmed cell death 1, PD-1), and CD154 (CD40 ligand, CD40L); secretes IL-2, IL-4 and IL-10; and helps B cells with Ab production [176]. These properties portray CXCR5+ Vγ9Vδ2 Tc as a memory Tc subset with B cell helper functions, analogous to follicular helper Tc (Tfh) [177]. Regulatory γδ Tc with immunosuppressive properties, able to produce transforming growth factor-beta (TGF-β) and IL-10, have also been described [152,156,178,179].

All these changes impose a large variability to the expression of cell surface and intracellular molecules. Among them, we will discuss only those evaluated in this study.

#### 4.8.1. CD3/TCR Complex

The human TCR-γδ is a protein complex composed of three heterodimers (TCR-γ/TCR-δ, CD3γ/CD3ε and CD3δ/CD3ε) and a ζ/ζ homodimer; the TCR-γδ heterodimer contains variable regions and lets for Ag recognition, while the other molecules are required for surface TCR expression and intracellular signaling [4,180].

In accordance to previous studies, we observed that the levels of CD3 expression were higher in γδ Tc as compared to αβ Tc, being lower in Vδ1 Tc than in Vδ2 Tc [181,182]. This could explain why the neoplastic Tc from patients with hepatosplenic γδ Tc lymphoma, which originate from the Vδ1 Tc subset, have been reported as having abnormally low levels of CD3 expression, as compared to those found in γδ Tc from “normal” and “reactive” PB samples (expressing mostly Vδ2) [183]. Differences in CD3 expression may influence Tc reactivity and the threshold for Tc activation after Ag recognition [182].

Concerning the phenotypic characterization of γδ Tc for the expression of different types of TCR-γδ, it is an opportune place to mention that there are many clones of anti-human “pan” TCR-γδ mAbs, and that some of them may not be able to identify every γδ Tc. In accordance, it has been reported that some anti-TCR-γδ clones may miss specific γδ Tc subsets, as identified by anti-Vδ1 (e.g., clones TS8.2 and R9.12) and anti-Vδ2 (e.g., clones B6 and Immu389) mAbs, respectively [184]. For instance, among three “pan” TCR-γδ mAbs tested by Wistuba-Hamprecht et al. (clone B1, APC; clone B1.1, PE; and clone 11F2A, PE-Cy7- and FITC), only one (clone 11F2A, unconjugated) was found to identify all Vδ1 and Vδ2 Tc [184]. We ourselves observed interferences when the “pan” anti-TCR-γδ (clone Immu510; PC5.5; BC/IOT) and the anti-Vδ1 (clone TS8.2; FITC; Endogen) were used in the same tube, although this interference did not prevent the quantification of Vδ1 Tc (Figure 1). A possible explanation could be steric hindrance effects because the epitopes recognized by these clones might be in proximity [184,185].

#### 4.8.2. CD5 Signaling Molecule

CD5, a transmembrane protein that associates with the CD3/TCR complex, and is expressed in most mature Tc, has been shown to regulate negatively TCR-mediated signaling, and to protect against autoimmunity [186]; however, high levels of CD5 expression may render Tc less able to recognize and eliminate malignant or viral-infected cells [187].

Previous studies have showed that the levels of CD5 on γδ Tc are lower than that observed in αβ Tc, and that CD5 negative γδ Tc are CTL [188]. In addition, it has been found that Vδ2 Tc are mainly CD5+, CD28+ and CD57-, whereas Vδ1 Tc tend to be CD5-/+low, CD28- and CD57+ [189]. In line with these observations, we found that the levels of CD5 expression were lower in γδ Tc as compared to αβ Tc, and lower in Vδ1 than in Vδ2 Tc, a larger fraction of CD5- cells being observed in the Vδ1 Tc compartment. Down regulation of CD5 may be a strategy adopted by tumor- or viral-specific CTL to optimize cytotoxicity and cytokine secretion [190].

Loss of CD5 expression has been considered an aberrant immunophenotypic feature, useful for identifying the neoplastic Tc, in the PB and in the involved tissues [191]. However, this characteristic may only traduce a terminal differentiation of the neoplastic Tc into CTL, as occurs in γδ Tc LGL leukemia [23], cutaneous γδ Tc lymphoma [192] and intestinal γδ Tc lymphoma [193]; or a premature loss of CD5 expression in activated, functionally-immature, cytotoxic, neoplastic Tc, as happens in hepatosplenic γδ Tc lymphoma [48].

#### 4.8.3. CD8 Accessory Molecule

The CD8 molecule is expressed on most CD8+ αβ Tc mainly as a CD8αβ heterodimer, and it acts as the co-receptor for MHC class I molecules.

Previous studies have reported that 20%–30% of peripheral blood γδ Tc are CD8+low, [188,194] expressing CD8α and CD8β polypeptides in all possible combinations, with the largest proportion of cells (44% ± 17%) exhibiting CD8ββ homodimers and smaller fractions expressing CD8αα homodimers (14% ± 12%) or CD8αβ heterodimers (14% ± 12%, and that the mean fluorescence intensity of CD8 expression is much lower in CD8αα and CD8ββ γδ Tc [181]. One study showed that CD8αβ+ γδ Tc were enriched within the gut mucosa comparatively to the PB; they expressed cytotoxic cell-associated markers, such as the CD56 adhesion molecule; and cytotoxic granule proteins, such as granzyme B and perforin; they produced mainly IFN-γ and TNF-α, and, in the PB, they were mostly Vδ1 [195]. This could explain why we observed that in nearly half of the normal PB samples analyzed, part of the Vδ1, but not of Vδ2 Tc, had high and homogeneous CD8 expression, at levels that approximated to those observed in CD8+high αβ Tc. The significance of this γδ Tc population needs to be clarified.

The precise functional role of the CD8αα and CD8ββ co-receptors is not known. Interestingly, both CD8αα and CD8αβ have been reported to bind to MHC class I and class I-like molecules, but CD8αβ was found to be more effective in promoting TCR activation [196]. In addition, CD8 has been suggested to play a role in TCR-independent immune responses of γδ Tc and NK cells [197].

During Ag recognition, both CD8αβ and CD8αα strengthen the TCRαβ binding to the MHC-peptide complex [198,199]. Nevertheless, efficient recruitment of CD8 molecules into lipid rafts, wherein they associate with CD3, TCR and p56(lck), seems to depend on CD8β chains [200,201]. Besides, CD8αα molecules have differential ability to bind distinct MHC (HLA in humans) class I molecules [202]. For instance, they bind normally to the non-classical MHC class I molecule HLA-G, but only weakly to HLA-E. Interestingly, HLA-G is mainly expressed in the placenta where it has been shown to inhibit trophoblast cells lysis by decidua NK cells [203], whereas HLA-E binds to the conserved leader peptides of a range of MHC class I molecules, and mediates protection from cytolysis by interacting with inhibitory CD94/NKG2 receptors expressed on NK cells and CTL, including γδ Tc [204]. It has also been proposed that CD8 expressed on γδ Tc might interact with MHC-like molecules (e.g., CD1, MICA, MICB) [181]. More recently, evidence has been provided that CD8αα homodimer suppresses Tc activation [205].

#### 4.8.4. CD28 Costimulatory Molecule

Activation of αβ Tc is induced primarily by signals generated by the specific interaction of a TCR with an Ag bound to the MHC molecules on APC. The major second signal needed for Tc activation is produced by the interaction of the CD28 molecule expressed in naïve and central memory Tc with their natural ligands, CD80 and CD86, located on APC, resulting in cell proliferation, cytokine production and effector functions [206,207]. CD28 is also constitutively expressed in naïve and central memory γδ Tc, and CD80/CD86-CD28 costimulatory signals were shown to control the survival and proliferation of γδ Tc via IL-2 production [208,209,210].

After Tc activation, some Tc lose CD28 expression, and chronic Ag stimulation leads to gradual accumulation of CD28 negative Tc [211,212]. CD28 negative Tc are Ag-experienced and Ag-specific, highly differentiated, effector Tc, with cytotoxic or immunosuppressive capacities; they are characterized by shortened telomeres and gain of CD57 expression, and they play significant roles in inflammatory conditions, infections and cancers [213].

Many age-associated changes have been described in αβ Tc, including the accumulation of terminally differentiated CD28 negative αβ Tc, and they have been attributed mainly to a latent infection with CMV [214]. Contrarily to what happens (and we observed) in αβ Tc, we found no significant correlation between the fraction of CD28- γδ Tc and age. This is agreement with that reported by Tan et al., who found that the proportion of terminally differentiated CD28-CD27- α/β Tc was significantly higher in the elderly compared to the young, but the fraction of CD28-CD27- Vδ2 Tc did not differ significantly between these age groups [215].

#### 4.8.5. CD16 and CD56—Cytotoxic-Cell-Associated Molecules

As NK cells, some γδ Tc do express CD16 (Fc gamma receptor type III, FcγRIII), which binds to the fragment of IgG with low affinity and leads to antibody-dependent cellular cytotoxicity (ADCC) [216]. Upon stimulation with non-peptide Ags (e.g., phosphoantigens), Vγ9Vδ2 Tc upregulate CD16 expression and produce large amounts of IFN-γ and TNF-α, and Vγ9Vδ2 Tc having high levels of CD16 are NKG2A/CD94+ perforin+ activated/memory cells [217,218]. Angenili et al. discriminated two populations of effector memory Vδ2 Tc: CD16 negative Vδ2 T_EM_ cells, expressing high levels of chemokine receptors, but low levels of perforin and of natural killer cell receptors (NKR); and CD16 positive Vδ2 T_EMRA_ cells, expressing several NKR and having high amounts of perforin, but low levels of chemokine receptors [218]. Whereas the former produced large amounts of IFN-γ and TNF-α in response to phosphoantigens, the later were refractory to phosphoantigen stimulation but reacted to activation via CD16 (FcγRIII) and were highly cytotoxic against tumor cells [217,218]. In addition, it was demonstrated that late-differentiated Vδ2 negative γδ Tc from CMV-seropositive individuals are CD16+high, and have intrinsic ADCC potential; CD16+ γδ Tc produced IFN-γ when incubated with IgG-opsonized CMV viruses, and IFN-γ production was enhanced by IL-12 and IFN-α, two cytokines produced during CMV infection, and conferred to γδ Tc the ability to inhibit CMV multiplication in vitro [219].

The neural cell adhesion molecule (NCAM, CD56), a cell adhesion molecule of the Ig-superfamily expressed on the surface of neurons and glial cells, has also been shown to be expressed on NK cells, and on Tc populations, including a fraction of γδ Tc [66]. CD56 expression in Tc defines a commitment to a cytotoxic phenotype [220,221], and CD56+ Vδ2 Tc have been characterized as having potent cytotoxic activity, being capable of killing tumor cells [174].

## 5. Take-Home Messages

Message 1: Due to the high biological variation, caution should be taken when interpreting quantitative and qualitative changes in circulating γδ Tc from individual subjects, which should always be read in the clinical context, to be considered, or not, pathological. Bearing in mind this variation and the difficulties in obtaining reference intervals for γδ Tc, we propose to use the minimum and maximum values found in healthy individuals as mere indicators of a possible pathological condition that needs to be further investigated.

Message 2: To facilitate comparisons between studies and to avoid bias related to the representation of other cell populations, we recommend expressing the Vγ and Vδ families and γδ Tc subsets as percentages of γδ Tc, and in absolute cell numbers, preferentially determined with single platform methods.

Message 3: Given the relatively scarce number of studies performed, the small size of the samples in most studies performed and/or missing or incomparable data, further investigations are needed to better evaluate the impacts of race, ethnicity and gender on γδ Tc and their subsets. With the information available, in young and middle-aged Caucasian European adults, we propose considering an increase of absolute and relative frequencies of γδ Tc if they exceed 300 cells/µL or 15% of the circulating Tc, and an increase in the absolute counts of Vδ2 and Vδ1 γδ Tc if they exceed 250 and 50 cells/µL, respectively.

Message 4: Age has a major impact on γδ Tc and their subsets: Vγ9Vδ2 Tc expressing a semi-invariant TCR are the predominant γδ Tc population in the PB from second-trimester fetuses, whereas Vδ1 Tc are overrepresented in the cord blood from neonates. Vγ9Vδ2 Tc increase from birth to about 10 years of age, accounting for the majority of circulating γδ Tc in young and middle-aged adults. Then, a progressive decrease of Vγ9Vδ2 occurs; these changes occur latterly and more slowly in females than in males, and are more evident in the elderly. Concomitantly, there is a shift from naïve to more differentiated effector γδ Tc phenotypes, which is more pronounced in Vδ1 Tc.

Message 5: In addition to an increase in Vδ1 Tc in the uterine decidua, changes occurring in peripheral blood γδ Tc and their subsets during healthy pregnancy probably contribute to fetal-maternal tolerance. Some of the reported variations include a diminished representation of Vγ9Vδ2 Tc, with increased proportions of other γδ Tc subsets, such as Vγ4Vδ1. However, considering the limited number of studies performed, additional investigation is needed on this subject.

Message 6: CMV infection has a substantial impact on γδ Tc and their subsets, affecting mainly Vδ2 negative γδ Tc, and contributing to many of the changes observed with aging; it may also explain some of the differences observed between races and ethnicities. Other HHVs may also contribute to modeling the γδ Tc repertoire, but their role needs further investigation.

Message 7: Among the medications influencing γδ Tc are bisphosphonates, largely used to treat osteoporosis, and statins, commonly used as anti-lipemic drugs. The dietary content of alkylamines may also impact γδ Tc.

Message 8: Gamma delta and αβ Tc share virtually all cell molecules, except the type of TCR. Upon antigen recognition, both Tc types express activation related markers, progress through sequential differentiation stages and may have diverse types of functional polarization, producing different sets of cytokines. As a consequence, and depending on the circumstances, both may exhibit different functions, acting as APC; helping B cells for antibody production; suppressing the immune responses or promoting the inflammatory responses; and eventually converting into cytotoxic Tc. The distinct immunophenotypes observed in γδ and αβ Tc, and in γδ Tc subsets, may traduce diverse activation and differentiation stages and/or distinct functional polarization, rather than being lineage related.

## 6. Conclusions

In summary, γδ Tc found in human PB are influenced by the race/ethnicity; age; gender; special periods of life, such as menarche, menstrual cycle, pregnancy and menopause; and by present and past immunological experiences, infections, diet, medications and previous and concomitant diseases; additionally, major immunophenotypic differences are observed between αβ and γδ Tc, and between γδ Tc subsets, which may result from different maturation/differentiation/activation stages and may account for distinct functional properties. 

Gamma/delta Tc and their subsets are frequently studied in the context of a wide range of pathological situations, and for the investigation of patients with cytopenias or suspected of having Tc LPD. However, due to the high variation observed, caution should be taken in interpreting the clinical significance of finding “abnormal levels” and/or “phenotypically abnormal” γδ Tc populations in individual patients.

Population-based studies using optimized multicolor FCM panels are required to define the reference intervals for the γδ Tc subsets and their differentiation and activation stages, accordingly to the demographic characteristics, and the influence of specific factors.

## Figures and Tables

**Table 1:** Hematological counts and percentages and absolute numbers of total peripheral blood γδ Tc in the study population of healthy adults.

Method (Equipment)	Parameter	Median (Min –Max)	Mean ± SD
Automated hematology analyzer (Coulter LH750)	Hemoglobin (g/dl)	14.4 (11.8–16.9)	14.2 ± 1.3
	Platelets (×10^9^/L)	207 (133–332)	225 ± 56
	WBC (cells/μL)	6700 (4800–9800)	6927 ± 1362
	Neutrophils (% WBC)	58.1 (44.1–78.8)	58.7 ± 7.7
	Neutrophils (cells/μL)	4034 (2506–7092)	4097 ± 1121
	Lymphocytes (% WBC)	30.8 (18.3–47.8)	30.6 ± 7.2
	Lymphocytes (cells/μL)	2087 (1122–3704)	2102 ± 601
Flow cytometry (BD FACS Canto II)	Tc (% Lymphocytes)	72.8 (57.4–85.6)	72.8 ± 6.9
	γδ Tc (% Tc)	4.3 (1.2–15.4)	5.0 ± 3.6
	Vδ1 Tc (% γδ Tc)	24.6 (3.5–65.7)	27.4 ± 16.1
	Vδ2 Tc (% γδ Tc)	66.4 (15.7–96.0)	60.0 ± 22.5
	Vγ9 Tc (% γδ Tc)	69.1 (25.7–96.5)	66.3 ± 20.5
	Vδ2/Vδ1 ratio	2.7 (0.3–27.7)	4.1 ± 5.4
Dual platform	Tc (cells/μL)	1547 (782–2356)	1533 ± 454
	γδ Tc (cells/μL)	63 (9–253)	78 ± 60
	Vδ1 Tc (cells/μL)	16 (2–75)	23 ± 19
	Vδ2 Tc (cells/μL)	39 (6–243)	63 ± 62
	Vγ9 Tc (cells/μL)	40 (7–247)	68 ± 62

Abbreviations: Tc, T cells. See Table S1 for information concerning testing for normal distribution and identification of outliers.

## Supplementary Materials

Supplementary material is available online at https://www.mdpi.com/2073-4409/9/3/729/s1. Table S1: Evaluation of the normality of distributions using the D’Agostino–Pearson and Kolmogorov–Smirnov tests and identification of outliers using the Tukey test. Table S2. Percentages of CD5, CD8, CD16, CD56 and CD28+ cells among peripheral blood γδ Tc, Vδ1+ and Vδ2+ γδ Tc, in the study population of healthy adults. Table S3. Percentages and numbers of peripheral blood γδ Tc and γδ Tc subsets accordingly to the age in the study population of healthy adults. Table S4. Percentages and numbers of peripheral blood γδ Tc and γδ Tc subsets accordingly to the gender in the study population of healthy adults. Table S5: Spearman’s rank correlations between the percentages and numbers of peripheral blood γδ Tc and γδ Tc subsets in the study population of normal healthy adults, by age and by gender. Table S6: Linear regression analysis between the percentages and numbers of peripheral blood γδ Tc and γδ Tc in the study population of normal healthy adults, by age and by gender. Table S7. Percentages and numbers of peripheral blood γδ Tc and γδ Tc subsets in the study population of healthy adults, accordingly to age and gender. Table S8. 95th reference intervals for the percentages and numbers of peripheral blood γδ Tc and γδ Tc subsets in the study population of healthy adults obtained with different statistic approaches. Figure S1: Relation between the logarithm of the percentages and numbers of peripheral blood γδ Tc and γδ Tc subsets in the study population of healthy adults, by ages and genders. Panel A: males + females; panel B: females; panel C: males. The Spearman’s rank correlation coefficients (R) and *p* values (*p*) are indicated inside the graphics.

## Abbreviations

ADCC: Antibody-dependent cellular cytotoxicity; Ag: Antigen; AIDS: Acquired immunodeficiency syndrome; APC: Allophycocyanin cyanine (fluorochrome); APC: Antigen-presenting cells; APC-H7: Allophycocyanin cyanine 7 (fluorochrome); B3A1: Butyrophilin 3A1; BC: Beckman Coulter; BD: Becton Dickinson; CB: Cord blood; CD: Cluster of differentiation; CDR3, complementary determining region 3; CD40L: CD40 ligand (CD154); CLSI: Clinical and Laboratory Standards Institute; CMV: Cytomegalovirus (HHV-5); CNS: Central nervous system; CTL: Cytotoxic T lymphocytes; CXCR5: CX-chemokine receptor type 5 (CD185); D’A-PT: D’Agostino–Pearson test (for normality) (statistic); EBV: Epstein Barr Virus (HHV-4); FCM: Flow cytometry; FcγRIII: Fc gamma receptor type III, low affinity Fc receptor for IgG; FDPS: Farnesyl diphosphate synthase; FITC: Fluorescein isothiocyanate (fluorochrome); FSC: Forward scatter; FT: F test (for variances) (statistics); HBV: Hepatitis B virus; HCV: Hepatitis C virus; HHV: Human herpes virus; HHV-1: Human herpes virus-1 (HSV-1); HHV-2: Human herpes virus-2 (HSV-2); HHV-3: Human herpes virus-3 (HZV); HHV-4: Human herpes virus-4 (EBV); HHV-5: Human herpes virus-5 (CMV); HHV-6: Human herpes virus-6; HHV-7: Human herpes virus-7; HHV-8: Human herpes virus-8 (KZHV); HIV: Human immunodeficiency virus; HMB-PP: (E)-4-hydroxy-3-methyl-but-2-enyl pyrophosphate; HMG-CoA: 3-hydroxy-3-methylglutaryl-coenzyme A; HPVB19: Human parvovirus B19; HSP: Heat shock proteins; HSV-1: Herpes simplex virus-1; HSV-2: Herpes simplex virus-2; HTLV: Human T cell lymphoma/leukemia virus; ICOS: Inducible T-cell costimulatory molecule (CD278); IFN-γ: Interferon gamma; IL: Interleukin; IOT: Immunotech; IPP: Isopentenyl pyrophosphate; K3-EDTA: Tripotassium ethylenediaminetetraacetic acid; KO, Krome orange (fluorochrome); KST: Kolmogorov–Smirnov test (for normality); KZHV: Kaposi’s sarcoma-associated herpes virus (HHV-8); LGL: Large granular lymphocyte; LPD: Lymphoproliferative disorders; mAb: Monoclonal antibody; MAIT: Mucosal associated invariant T cells; MedFI: Median fluorescence intensity; MHC: Major histocompatibility complex; MIC: MHC class I polypeptide-related chains; MIC-A: MHC class I polypeptide-related chain type A; MIC-B: MHC class I polypeptide-related chain type B; MIP-1α: Macrophage inflammatory protein-1 alpha: MWUT: Mann–Whitney U test (statistic); NA: Not applicable; NAT: Nucleic acid tests; NBP: Nitrogen-containing bisphosphonates; NCAM: Neural cell adhesion molecule; NCCLS: National Committee for Clinical Laboratory Standards; NK, Natural killer; NKG2D: Natural killer group 2 member D (killer cell receptor); NKR: Natural killer cell receptors; PB: Peripheral blood; PC5.5: PE-Cyanine 5.5 (fluorochrome); PC7: PE-Cyanine 7 (fluorochrome); PD-1: Programmed cell death 1, PD-1 (CD279); PE: Phycoerythrin (fluorochrome); PHA: Phytohemagglutinin (mitogen); PMT: Photomultiplier tubes; PTLS: Post-transplant lymphoproliferative syndrome; RAET1: Retinoic acid early transcripts; RANTES: Regulated on activation, normally T cell expressed or secreted; RAU: Recurrent aphthous ulceration; SD: Standard deviation; SRCT: Spearman rank correlation test; SSC: Side scatter; Tc: T cells; TCM: Central memory T cells; TCR: T-cell receptor; T_EM_: Effector memory T cells; T_EMRA_: Terminally differentiated effector T cells; Tfh: Follicular helper T cells; TGF-β: Transforming growth factor-beta; Th: T helper; Th1: T helper type 1; Th17: T helper type 17; Th2: T helper type 2; T_N_: Naïve T cells; TNF-α: Tumor necrosis factor alpha; Treg: Regulatory T cells; TT: Tukey test (statistic); ULBP: UL16 binding proteins; V450: Violet 450 (fluorochrome); VZV: Varicella Zoster Virus (HHV-3); WBC: White blood cells; WHO: World Health Organization; WSRT: Wilcoxon signed-rank (statistics).
