# Supplementary material for: Human Peripheral Blood Gamma Delta T Cells: Report on a Series of Healthy Caucasian Portuguese Adults and Comprehensive Review of the Literature
Source: Cells. 2020 Mar 16;9(3):729. doi: 10.3390/cells9030729 (PMC7140678; doi:10.3390/cells9030729)
Supplement: Supplementary file 1 [file cells-09-00729-s001.pdf]

Title: Human Peripheral Blood Gamma Delta T Cells: Report on a Series of Healthy Caucasian Portuguese Adults and Comprehensive Review of the Literature

SUPPLEMENTARY MATERIAL

Table S1: Evaluation of the Normality of distributions using the D'Agostino-Pearson and Kolmogorov-Smirnov tests and identification of outliers using the Tukey test.

Table S2. Percentages of CD5, CD8, CD16, CD56, and CD28+ cells among peripheral blood γδ Tc, Vδ1+ and Vδ2+ γδ Tc in the study population of healthy adults.

Table S3. Percentages and numbers of peripheral blood γδ Tc and γδ Tc subsets accordingly to the age in the study population of healthy adults.

Table S4. Percentages and numbers of peripheral blood γδ Tc and γδ Tc subsets accordingly to the gender in the study population of healthy adults.

Table S5: Spearman's rank correlations between the percentages and numbers of peripheral blood γδ Tc and γδ Tc subsets in the study population of healthy adults, and the age, by gender.

Table S6: Linear regression analysis between the percentages and numbers of peripheral blood γδ Tc and γδ Tc in the study population of healthy adults, and the age, by gender.

Table S7. Percentages and numbers of peripheral blood γδ Tc and γδ Tc subsets in the study population of healthy adults, accordingly to the age, by gender.

Table S8. 95th reference intervals for the percentages and numbers of peripheral blood γδ Tc and γδ Tc subsets in the study population of healthy adults obtained with different statistic approaches.

Figure S1: Relation between the logarithm of the percentages and numbers of peripheral blood γδ Tc and γδ Tc subsets in the study population of healthy adults, and the age, by gender. Panel A: males + females; panel B: females; panel C: males. The Spearman's rank correlation coefficients (R) and p values (P) are indicated inside the graphics.

Table S1. Evaluation of the Normality of distributions using the D'Agostino-Pearson and Kolmogorov-Smirnov tests and identification of outliers using the Tukey test.

**Method (equipment)**	**Parameter**	**Tests for Normal distribution**			**Out**
		D'A-PT (P)	KST (P)	ND	TT
Blood cell counts	Hemoglobin (g/dl)	0.556	>0.100	Yes	0
	Platelets (x 10^9/L)	0.499	0.068	Yes	0
	WBC (cells/μl)	0.386	>0.100	Yes	0
	Neutrophils (% WBC)	0.570	>0.100	Yes	0
	Neutrophils (cells/μl)	0.179	>0.100	Yes	0
	Lymphocytes (% WBC)	0.735	>0.100	Yes	0
	Lymphocytes (cells/μl)	0.243	>0.100	Yes	1
T cell populations	Tc (% Lymphocytes)	0.807	>0.100	Yes	0
	γδ Tc (% Tc)	**0.002**	0.070	**No**	2
	Vδ1 Tc (% γδ Tc)	0.192	>0.100	Yes	0
	Vδ2 Tc (% γδ Tc)	0.272	>0.100	Yes	0
	Vγ9 Tc (% γδ Tc)	0.335	>0.100	Yes	0
	Vδ2/Vδ1 ratio	**<0.001**	**<0.001**	**No**	2
T cell counts	Tc (cells/μl)	0.05	>0.100	Yes	1
	γδ Tc (cells/μl)	**0.004**	**0.002**	**No**	1
	Vδ1 Tc (cells/μl)	**<0.001**	**<0.001**	**No**	4
	Vδ2 Tc (cells/μl)	**<0.001**	**0.001**	**No**	2
	Vγ9 Tc (cells/μl)	**<0.001**	**0.002**	**No**	0

Peripheral blood samples from 30 healthy Caucasian European Portuguese adult individuals (blood donors), 18 males and 12 females, with median age of 47 years, ranging from 26 to 66 years (mean ± standard deviation of 48 ± 14 years).
Abbreviations: ND, Normal distribution; Out, number of outliers; Tc, T cells; WBC, white blood cells.
D'A-PT: D'Agostino-Pearson test (P value for Normal distribution); KST: Kolmogorov-Smirnov test (P value for Normal distribution); TT: Tukey test.

Table S2. Percentages of CD5, CD8, CD16, CD56, and CD28+ cells among peripheral blood γδ Tc, Vδ1+ and Vδ2+ γδ Tc in the study population of healthy adults.

Parameter	γδ Tc	Vδ1 Tc	Vδ2 Tc	Vδ1 vs. Vδ2 Tc P (MWUT)
% CD5+ cells	93.2 (57.1 – 98.9)<br>88.2 ± 11.7	83.8 (20.5 – 99.0)<br>77.2 ± 22.9	98.7 (84.4 – 99.9)<br>96.8 ± 4.3	**<0.001**
% CD8+ cells	28.6 (11.5 – 66.9)<br>31.6 ± 13.0	44.9 (10.9 – 89.9)<br>46.5 ± 21.7	17.2 (9.4 – 41.4)<br>20.3 ± 9.1	**<0.001**
% CD16+ cells	22.3 (6.4 – 72.8)<br>27.5 ± 18.0	12.6 (0.1 – 73.5)<br>18.5 ± 17.5	15.0 (3.4 – 84.7)<br>27.1 ± 23.9	0.214
% CD56+ cells	39.9 (12.0 – 71.1)<br>40.2 ± 15.7	34.8 (3.3 – 79.5)<br>35.6 ± 21.6	46.0 (13.5 – 74.7)<br>43.8 ± 17.6	0.104
% CD16/CD56+ cells	45.3 (12.3 – 77.3)<br>46.8 ± 18.2	45.9 (10.8 – 87.0)<br>44.0 ± 22.2	52.5 (18.2 – 91.1)<br>51.7 ± 19.7	0.156
% CD28+ cells	41.6 (9.0 – 80.6)<br>45.5 ± 21.5	17.5 (4.4 – 73.0)<br>23.4 ± 18.7	66.4 (2.7 – 94.0)<br>59.3 ± 25.4	**<0.001**

Abbreviations: MWUT: Mann-Whitney U test; P, p value; Tc, T cells. Results are presented as median, (minimum – maximum) values, and mean ± SD.

Table S3. Percentages and numbers of peripheral blood γδ Tc and γδ Tc subsets accordingly to the age in the study population of healthy adults.

	20 to 45 years (n=13)	46 to 70 years (n=17)	P (MWUT)
**Gender**<br>Males: females (ratio)	8:5 (1.6)	10:7 (1.4)	**NA**
**γδ Tc (% Tc)**	5.7 (1.9 - 13.6)<br>6.5 ± 3.3	3.0 (1.2 - 15.4)<br>3.8 ± 3.3	**0.010**
**Vδ1 Tc (% γδ Tc)**	24.2 (3.5 - 58.1)<br>27.5 ± 16.6	29.6 (6.2 - 65.7)<br>27.2 ± 16.1	0.984
**Vδ2 Tc (% γδ Tc)**	68.4 (15.7 - 96.0)<br>62.7 ± 22.2	60.0 (19.6 - 93.4)<br>57.9 ± 23.2	0.477
**Vγ9 Tc (% γδ Tc)**	72.9 (27.9 - 96.5)<br>70.8 ± 18.3	61.7 (25.7 - 95.1)<br>62.8 ± 21.9	0.267
**Vδ2/Vδ1 ratio**	2.7 (0.3 - 27.7)<br>4.8 ± 7.2	2.0 (0.5 - 15.1)<br>3.7 ± 3.7	0.818
**γδ Tc (cells/μl)**	76 (39 - 217)<br>102 ± 55	45 (9 - 253)<br>60 ± 59	**0.014**
**Vδ1 Tc (cells/μl)**	17 (8 - 75)<br>32 ± 25	15 (2 - 35)<br>15 ± 10	0.065
**Vδ2 Tc (cells/μl)**	56 (17 - 220)<br>80 ± 60	19 (6 - 243)<br>49 ± 62	**0.035**
**Vγ9 Tc (cells/μl)**	73 (30 - 221)<br>90 ± 58	26 (7 - 247)<br>52 ± 62	**0.008**

Abbreviations: MWUT: Mann-Whitney U test; NA, not applicable; P, p value; Tc, T cells. Results are presented as median, (minimum – maximum) values, and mean ± standard deviation.

Table S4. Percentages and numbers of peripheral blood γδ Tc and γδ Tc subsets accordingly to the gender in the study population of healthy adults.

T cell population	Males (n=18)	Females (n=12)	P (MWUT)	P (FT)
**Age (years)**	46 (26 - 68)<br>47 ± 13	52 (22 - 66)<br>49 ± 15	NA	NA
**γδ Tc (% Tc)**	2.9 (1.2 - 15.4)<br>5.1 ± 4.5<br>[20.2]	4.6 (2.2 - 7.9)<br>4.8 ± 1.4<br>[2.0]	0.352	**<0.001**
**Vδ1 Tc (% γδ Tc)**	27.4 (3.5 - 65.7)<br>29.5 ± 17.5<br>[305.7]	21.0 (11.9 - 58.1)<br>24.1 ± 13.9<br>[194.6]	0.397	0.450
**Vδ2 Tc (% γδ Tc)**	57.7 (23.2 - 96.0)<br>58.4 ± 21.6<br>[466.4]	72.0 (15.7 - 84.4)<br>62.4 ± 24.7<br>[607.9]	0.395	0.604
**Vγ9 Tc (% γδ Tc)**	69.1 (26.6 - 96.5)<br>67.1 ± 18.3<br>[334.2]	72.4 (25.7 - 93.8)<br>65.1 ± 24.2<br>[586.8]	0.966	0.288
**Vδ2/Vδ1 ratio**	2.0 (0.5 - 27.7)<br>4.5 ± 6.8<br>[46.0]	3.5 (0.3 - 7.0)<br>3.6 ± 2.3<br>[5.3]	0.446	**0.001**
**γδ Tc (cells/µl)**	49 (9 - 253)<br>79 ± 75<br>[5658]	74 (42 - 134)<br>78 ± 29<br>[837]	0.220	**0.003**
**Vδ1 Tc (cells/µl)**	15 (1 - 75)<br>23 ± 23<br>[513]	16 (6 - 61)<br>22 ± 14<br>[203]	0.602	0.122
**Vδ2 Tc (cells/µl)**	31 (6 – 24)<br>63 ± 75<br>[5557]	55 (16 - 124)<br>61 ± 39<br>[1487]	0.391	**0.031**
**Vγ9 Tc (cells/µl)**	38 (7 - 247)<br>71 ± 75<br>[5606]	57 (16 - 123)<br>64 ± 39<br>[1542]	0.573	**0.034**

Abbreviations: FT: F test, for equal variances; MWUT: Mann-Whitney U test NA, not applicable; P, p value; Tc, T cells. Results are presented as median, (minimum – maximum), mean ± SD and [variance].

Table S5. Spearman's rank correlations between the percentages and numbers of peripheral blood γδ Tc and γδ Tc subsets in the study population of healthy adults, and the age, by gender.

T cell population	Study population (n=30)	Females (n=12)	Males (n=18)
γδ Tc (% Tc)	r = -0.396<br>**p = 0.031**	r = -0.389<br>p = 0.212	r = -0.476<br>**p = 0.046**
Vδ1 Tc (% γδ Tc)	r = -0.143<br>p = 0.450	r = -0.389<br>p = 0.212	r = 0.024<br>p = 0.925
Vδ2 Tc (% γδ Tc)	r = 0.021<br>p = 0.912	r = 0.224<br>p = 0.484	r = -0.036<br>p = 0.888
Vγ9 Tc (% γδ Tc)	r = 0.0142<br>p = 0.940	r = 0.095<br>p = 0.770	r = 0.061<br>p = 0.810
Vδ2 / Vδ1 ratio	r = 0.106<br>p = 0.576	r = 0.378<br>p = 0.225	r = -0.046<br>p = 0.857
γδ Tc (cells/μl)	r = -0.392<br>**p = 0.032**	r = -0.053<br>p = 0.871	r = -0.613<br>**p = 0.007**
Vδ1 Tc (cells/μl)	r = -0.419<br>**p = 0.021**	r = -0.329<br>p = 0.296	r = -0.558<br>**p = 0.016**
Vδ2 Tc (cells/μl)	r = -0.276<br>p = 0.139	r = 0.165<br>p = 0.609	r = -0.577<br>**p = 0.012**
Vγ9 Tc (cells/μl)	r = -0.337<br>p = 0.069	r = 0.025<br>p = 0.940	r = -0.549<br>**p = 0.018**

Abbreviations: Tc, T cells.
The results are presented as Spearman's rank correlation coefficients (r) and p values.

Table S6. Linear regression analysis between the percentages and numbers of peripheral blood γδ Tc and γδ Tc subsets in the study population of healthy adults, and the age, by gender.

T cell population	Study population (n=30)	Females (n=12)	Males (n=18)
**γδ Tc (% Tc)**	R^2 = 0.08590<br>y = 8,5843 + -0,07582 x	R^2 = 0.1259<br>y = 6,3772 + **-0,03309** x	R^2 = 0.1074<br>y = 10,4174 + **-0,1130** x
Logarithmic Transformation	R^2 = 0.1413<br>log(y) = 0,9915 + -0,008300 x	R^2 = 0.04990<br>log(y) = 0,7580 + **-0,002028** x	R^2 = 0.2502<br>log(y) = 1,2285 + **-0,01436** x
**γδ Tc (cells/µl)**	R^2 = 0.07185<br>y = 134,1839 + -1,1748 x	R^2 = 0.02808<br>y = 93,1334 + **-0,3168** x	R^2 = 0.1135<br>y = 169,8364 + **-1,9431** x
Logarithmic Transformation	R^2 = 0.1715<br>log(y) = 2,2869 + -0,01102 x	R^2 = 0.02899<br>log(y) = 1,9496 + **-0,001759** x	R^2 = 0.3403<br>log(y) = 2,6344 + **-0,02003** x
**Vδ1 Tc (cells/µl)**	R^2 = 0.1884<br>y = 51.6875 + -0.6134 x	R^2 = 0.06641<br>y = 33.3499 + **-0.2400** x	R^2 = 0.2961<br>y = 67.3707 + **-0.9447** x
Logarithmic Transformation	R^2 = 0.2209<br>log(y) = 1,8224 + -0,01291 x	R^2 = 0.1065<br>log(y) = 1,5203 + **-0,005136** x	R^2 = 0.3493<br>log(y) = 2,1207 + **-0,02034** x
**Vδ2 Tc (cells/µl)**	R^2 = 0.01409<br>y = 87.9312 + -0.5334 x	R^2 = 0.01301<br>y = 47.3104 + 0.2874 x	R^2 = 0.04856<br>y = 122.5156 + **-1.2595** x
Logarithmic Transformation	R^2 = 0.09648<br>log(y) = 2,0729 + -0,01010 x	R^2 = 0.004351<br>log(y) = 1,6211 + **0,001401** x	R^2 = 0.2879<br>log(y) = 2,5161 + -0,02107 x
**Vγ9 Tc (cells/µl)**	R^2 = 0.02805<br>y = 104,2179 + -0,7585 x	R^2 = 0.003336<br>y = 57,1226 + 0,1482 x	R^2 = 0.07250<br>y = 143,2065 + **-1,5457** x
Logarithmic Transformation	R^2 = 0.1188<br>log(y) = 2,1632 + -0,01080 x	R^2 = 0.000002884<br>log(y) = 1.7209 + **0,00003312** x	R^2 = 0.2967<br>log(y) = 2,5864 + **-0,02100** x

Abbreviations: Tc, T cells.
Linear regression analysis: coefficients of determination (R2) and regression equations: y = a + bx and log(y) = a + bx, where “y” represents the immunological parameter, “x” represents age, “a” represents the intercept, and “b” represents the slope.

Table S7. Percentages and numbers of peripheral blood γδ Tc and γδ Tc subsets in the study population of healthy adults, accordingly to the age, by gender.

	20 to 40 years (n=13)				46 to 70 years (n=17)			
**T cells**	**Males (n=8)**	**Females (n=5)**	**P (MWUT)**	**P (FT)**	**Males (n=10)**	**Females (n=7)**	**P (MWUT)**	**P (FT)**
**Total γδ Tc (% Tc)**	7.9<br>(1.9 - 13.6)<br>7.4 ± 3.8<br>[14.7]	5.5<br>(2.2 - 7.9)<br>5.2 ± 2.0<br>[4.1]	0.222	0.236	2.1<br>(1.2 - 15.4)<br>3.3 ± 4.3<br>[18.6]	4.3<br>(3.4 - 6.1)<br>4.5 ± 0.8<br>[0.7]	**0.006**	**0.001**
**Vδ1 Tc (% γδ Tc)**	24.6<br>(3.5 - 55.4)<br>25.2 ± 17.0<br>[287.9]	21.3<br>(20.7 - 58.1)<br>30.4 ± 16.0<br>[257.3]	0.724	0.969	31.0<br>(6.2 - 65.9)<br>33.0 ± 18.0<br>[324.1]	13.0<br>(11.9 - 36.9)<br>19.6 ± 11.3<br>[128.5]	0.118	0.273
**Vδ2 Tc (% γδ Tc)**	66.4<br>(35.5 - 96.0)<br>63.1 ± 21.4<br>[458.4]	74.2<br>(15.7 - 76.2)<br>62.1 ± 26.0<br>[677.2]	0.622	0.611	54.8<br>(23.2 - 93.5)<br>54.6 ± 22.1<br>[489.3]	69.7<br>(19.6 - 84.4)<br>62.6 ± 25.7<br>[662.8]	0.329	0.504
**Vγ9 Tc (% γδ Tc)**	71.3<br>(46.0 - 96.5)<br>72.6 ± 16.0<br>[257.2]	79.8<br>(27.9 - 84.3)<br>68.0 ± 23.2<br>[538.8]	0.724	0.370	61.3<br>(26.6 - 95.1)<br>62.6 ± 19.5<br>[381.9]	61.7<br>(25.7 - 93.8)<br>63.0 ± 26.5<br>[704.7]	0.845	0.972
**Vδ2/Vδ1 ratio**	2.7<br>(0.6 - 27.7)<br>6.1 ± 9.0<br>[81.3]	3.5<br>(0.3 - 3.6)<br>2.6 ± 1.4<br>[2.1]	0.770	**0.003**	1.6<br>(0.5 - 15.1)<br>3.2 ± 3.4<br>[19.5]	4.5<br>(0.6 - 7.0)<br>4.3 ± 2.6<br>[7.0]	0.172	0.222
**Total γδ Tc (cells/µl)**	114<br>(39 - 217)<br>116 ± 63<br>[3997]	72<br>(50 - 134)<br>80 ± 32<br>[993]	0.524	0.197	25<br>(9 - 253)<br>49 ± 73<br>[5372]	77<br>(42 - 124)<br>76 ± 29<br>[861]	**0.019**	**0.037**
**Vδ1 Tc (cells/µl)**	17<br>(8 - 75)<br>35 ± 29<br>[813]	16<br>(15 - 61)<br>28 ± 20<br>[395]	0.943	0.507	11<br>(2 - 35)<br>14 ± 11<br>[129]	16<br>(6 - 29)<br>17 ± 7<br>[54]	0.435	0.512
**Vδ2 Tc (cells/µl)**	70<br>(33 - 220)<br>95 ± 68<br>[4660]	54<br>(17 - 111)<br>55 ± 35<br>[1249]	0.354	0.221	18<br>(6 - 243)<br>38 ± 72<br>[5247]	65<br>(16 - 124)<br>66 ± 43<br>[1835]	**0.064**	0.214
**Vγ9 Tc (cells/µl)**	98<br>(38 - 221)<br>107 ± 64<br>[4054]	58<br>(30 - 123)<br>61 ± 37<br>[1379]	0.171	0.314	21<br>(7 - 247)<br>42 ± 73<br>[54]	57<br>(16 - 118)<br>66 ± 44<br>[1894]	**0.064**	0.221

Abbreviations: MWUT, Mann-Whitney U test; FT, F test (for equal variances); P, p value; Tc, T cells. Results are presented as median (minimum – maximum) values, mean ± SD and [variance]

Table S8. 95th reference intervals for the percentages and numbers of peripheral blood γδ Tc and γδ Tc subsets in the study population of healthy adults obtained with different statistic approaches.

	Parametric 97.5th percentile method		Non-parametric percentile method		Percentile bootstrap method ("Robust Method")	
**T cell population**	**LL**	**UP**	**LL**	**UP**	**LL**	**UL**
**γδ Tc (% Tc)**<br>Yes (2) *	-2.0<br>(-3.9 to -0.1)	**12.0**<br>(10.1 to 13.8)	1.2	**15.4**	-3.4<br>(-5.6 to -0.9)	**11.6**<br>(8.8 to 14.3)
**Vδ1 Tc (% γδ Tc)**<br>No (0)	-4.3<br>(-12,8 to 4,2)	**59.0**<br>(50.5 to 67.5)	3.5	**65.7**	-8.6<br>(-18,0 to -0,9)	**59.5**<br>(48.3 to 69.3)
**Vδ2 Tc (% γδ Tc)**<br>No (0)	15.8<br>(3.9 to 27.7)	**104.0**<br>(99.2 to 116.0)	15.7	**96.0**	12.3<br>(1.6 to 29.2)	**110.1**<br>(99.4 to 119.2)
**Vγ9 Tc (% γδ Tc)**<br>No (0)	26.1<br>(15.4 to 36.9)	**106.4**<br>(95.7 to 117.2)	25.7	**96.5**	26.7<br>(13.3 to 39.2)	**112.8**<br>(102.2 to 120.6)
**γδ Tc (cells/µl)**<br>Yes (1)	-40<br>(-71 to -8)	**197**<br>(165 to 228)	9	**253**	-67<br>(-101 to -25)	**189**<br>(143 to 235)
**Vδ1 Tc (cells/µl)**<br>Yes (4)	-16<br>(-26 to -5)	**61**<br>(50 to 71)	1	**75**	-27<br>(-38 to -11)	**57**<br>(41 to 71)
**Vδ2 Tc (cells/µl)**<br>Yes (2)	-59<br>(-91 to -26)	**184**<br>(151 to 216)	6	**243**	-98<br>(-135 to -45)	**176**<br>(123 to 222)
**Vγ9 Tc (cells/µl)**<br>No (0)	-54<br>(-87 to -21)	**190**<br>(157 to 223)	7	**247**	-94<br>(-129 to -42)	**189**<br>(133 to 231)

Abbreviations: Tc, T cells. * Yes or No refers to presence or absence of suspected outliers, respectively (Tukey test). The number of outliers in indicated between brackets. Outliers were not excluded for analysis.

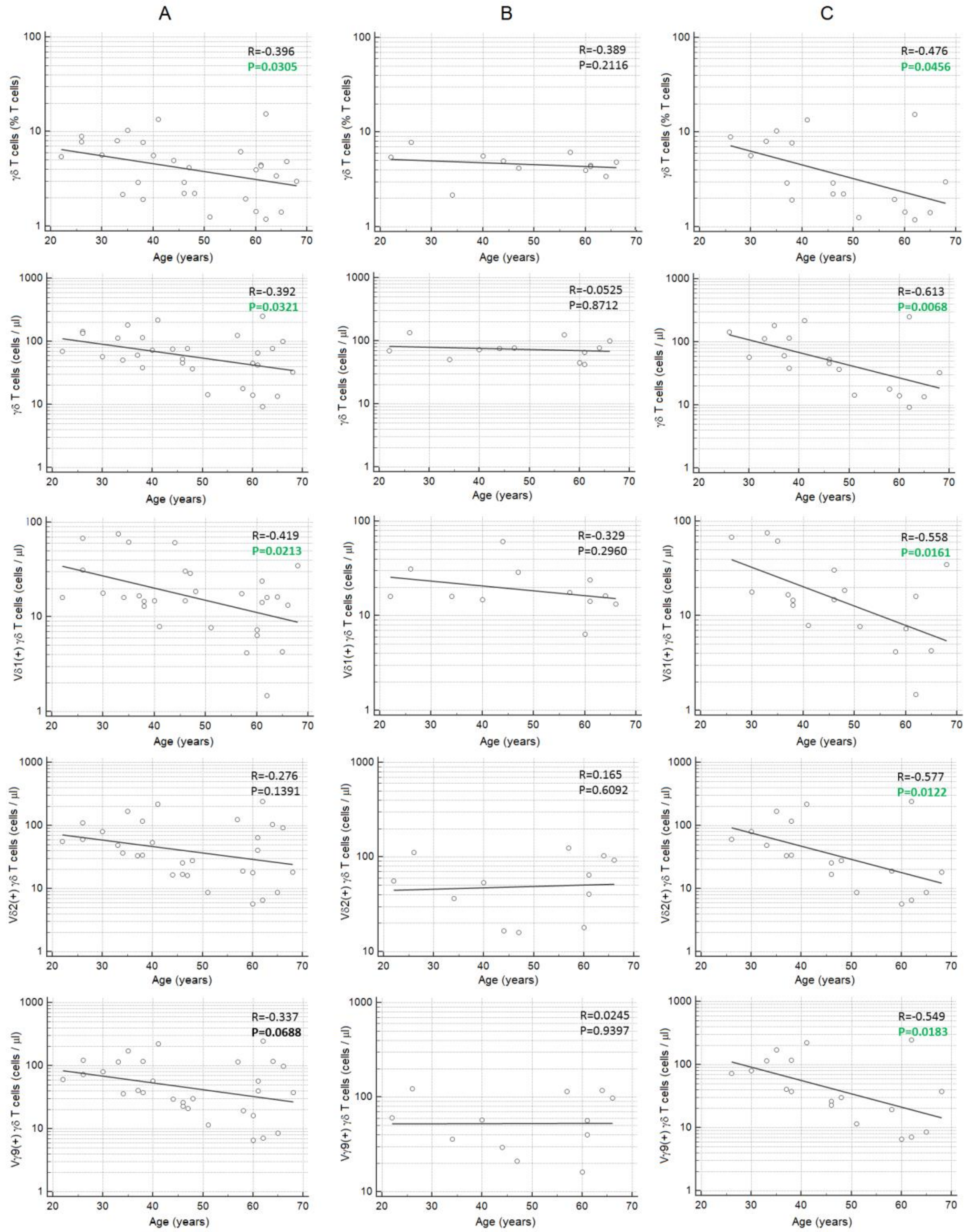

Figure S1. Relation between the logarithm of the percentages and numbers of peripheral blood γδ T cells and γδ T cell subsets in the study population of healthy adults, and the age, by gender. Panel A: males + females; panel B: females; panel C: males. The Spearman's rank correlation coefficients (R) and p values (P) are indicated inside the graphics.
